# Stockpiling by pups and self-sacrifice by their fasting mothers observed in birth to weaning serum metabolomes of Atlantic grey seals

**DOI:** 10.1038/s41598-020-64488-1

**Published:** 2020-05-04

**Authors:** David G. Watson, Patrick P. Pomeroy, Naser F. Al-Tannak, Malcolm W. Kennedy

**Affiliations:** 1 Strathclyde Institute of Pharmacy and Biomedical Sciences, University of Strathclyde, 161 Cathedral Street, Glasgow, G4 0RE Scotland UK; 2 Sea Mammal Research Unit, Scottish Oceans Institute, University of St Andrews, St Andrews, Fife, Scotland United Kingdom; 3 Department of Pharmaceutical Chemistry, Faculty of Pharmacy, Kuwait University, P.O. Box 23924, Safat, 13110 Kuwait City Kuwait; 4 Institute of Biodiversity, Animal Health & Comparative Medicine, Graham Kerr Building, College of Medical, Veterinary and Life Sciences, University of Glasgow, Glasgow, G12 8QQ Scotland UK

**Keywords:** Biochemistry, Physiology, Zoology

## Abstract

During the uniquely short lactations of true seals, pups acquire a greater proportion of maternal body resources, at a greater rate, than in any other group of mammals. Mothers in many species enter a period of anorexia but must preserve sufficient reserves to fuel hunting and thermoregulation for return to cold seas. Moreover, pups may undergo a period of development after weaning during which they have no maternal care or nutrition. This nutritionally closed system presents a potentially extreme case of conflict between maternal survival and adequate provisioning of offspring, likely presenting strains on their metabolisms. We examined the serum metabolomes of five mother and pup pairs of Atlantic grey seals, *Halichoerus grypus*, from birth to weaning. Changes with time were particularly evident in pups, with indications of strain in the fat and energy metabolisms of both. Crucially, pups accumulate certain compounds to levels that are dramatically greater than in mothers. These include compounds that pups cannot synthesise themselves, such as pyridoxine/vitamin B6, taurine, some essential amino acids, and a conditionally essential amino acid and its precursor. Fasting mothers therefore appear to mediate stockpiling of critical metabolites in their pups, potentially depleting their own reserves and prompting cessation of lactation.

## Introduction

Reproduction by mammals is both energy- and resource-intensive. Gestation and lactation present a considerable strain on maternal metabolism, body stores, mobility, can increase vulnerability to predation, and compromise future fitness^1–4^. The lactation strategies of mammals vary dramatically from long lactation periods that accommodate the growth and development of the highly altricial neonates (born at an early stage of development) of marsupials to the generally shorter lactations in eutherian (‘placental’) mammals that produce neonates at more advanced stages of development and growth^4–6^. No eutherian gives birth to neonates as extremely altricial as do marsupials, but they nevertheless differ considerably in the stage of development at birth that in turn approximately determines the length of lactation^4^. At the extremes among eutherians are bears, which have altricial neonates and long lactations, to the many groups that have precocial offspring and shorter lactations relative to body size, such as the bovids and equids^4,7^. But it is the true seals (Phocidae) that exhibit the shortest (four days in hooded seals) and most intense lactations, with extremely rapid weight gain in their pups^8^. Remarkably, despite these extreme disparities, the bears and the true seals are both within the same infraorder of the Carnivora, the Arctoidea^9–11^.

All species of oceanic mammal (cetaceans, pinnipeds, sirenians) give birth to precocial young. Some do so at sea, some on land, and some on ice. Of those that give birth on land or ice, there are three groups, the true seals (Phocidae), the Otariidae (fur or eared seals, sea lions), and the Odobenidae (walrus)^12^. The true seals are unusual in that mothers in half of the species do not feed during lactation, during which time their pups may double their body weights^13–17^, and mothers usually leave their offspring abruptly at weaning^8^. Mothers, meanwhile, must retain sufficient body resources, blubber in particular, to fuel their hunting activities and provide sufficient insulation against the cold of polar and subpolar seas. Pups of many species of seal undergo a period of post-weaning development on land with no further maternal support, so the accumulation of sufficient reserves by pups prior to weaning is critical. The duration of the lactation period, its draw on maternal resources, and the nutrients that pups accumulate therefore represent a strategy of fine balance between maternal survival and adequate provisioning of offspring.

We have previously followed metabolomic changes in the milk of mother seals from birth to weaning. Some of these changes potentially indicate the onset of stress in maternal fat metabolism^18^. We speculated that compounds indicating metabolic stress may leak into milk from the mother’s circulation, although they could arise from the mammary gland itself. To distinguish systemic versus mammary sources of these compounds, and to better understand the significance of the observations in the milk metabolome for the mother and pup, we followed metabolomic changes in the peripheral blood of both mothers and pups from birth to weaning. We did this in Atlantic grey seals, *Halichoerus grypus*, whose females endure a fast while lactating for about twenty days before weaning their pups and returning to sea^8,16^. Following the mother’s departure, grey seal pups have been reported to remain on land for 10–40 days, during which they obtain no nutrition, before they are themselves seaworthy^19^.

The brief lactation period, the strain on the mother’s energy balance, and the rapid development of a newborn pup with only three weeks of nutrition followed by up to a month of fasting pose a number of intriguing questions. How does a seal pup’s metabolism change from birth to weaning? How do pups prepare metabolically for their post-weaning fast on land? How does a mother seal’s metabolism alter during her fast? Are there discernible indicators of a mother reaching the point where her reserves are becoming depleted and she must return to feeding and abandon her pup?

We found that there are distinct alterations in the serum metabolomes of both mothers and pups between the onset of lactation and weaning, overlaying a remarkable metabolic stability in the mothers. We also found an overabundance of certain compounds in pup sera relative to maternal sera. We suggest that this disparity may represent stockpiling of essential compounds by pups in preparation for their period of post-weaning development.

## Results and Discussion

### Comparative changes in the serum metabolomes of pups and their mothers

**Figure 1:**
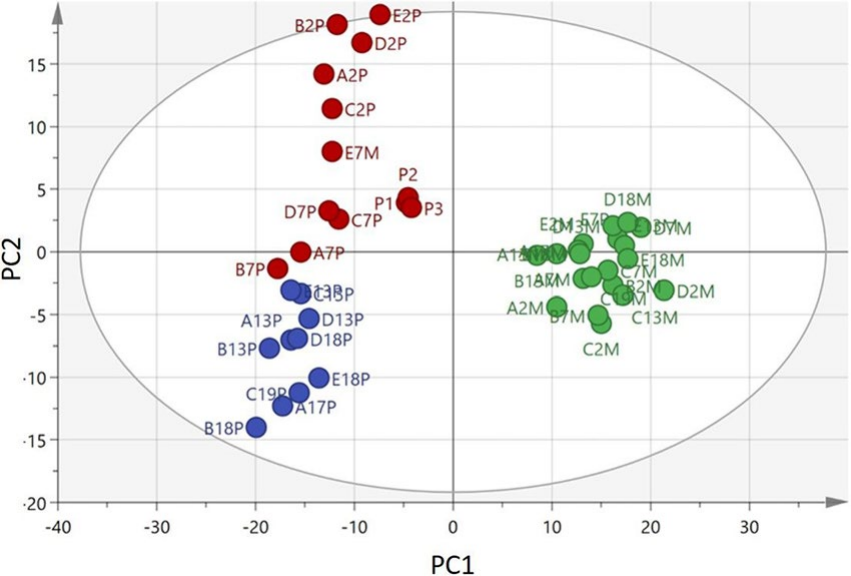
PCA analysis of the metabolome of serum samples from Atlantic grey seal mothers and pups based on 615 features in positive and negative ion mode. The pooled control samples from pups (P1, P2, and P3) overlap in the middle of the plot indicating consistency in instrument reading throughout the analytical run covering all the individual samples. Data point codes – the initial letter is the seal mother and pup pair (**A to E**), the numeral is the days after birth, then ‘M’ for mother and ‘P’ for pup. Green – mothers. Red – pups sampled on days 2 and 7. Blue – pups sampled on days 13 or 18.

Serum samples varied dramatically in turbidity with time, particularly in pups, presumably due to fats and (proteo)lipid micelles in circulation (Supplementary, Fig. S1). We first carried out a broad metabolomic screen that revealed wide separation between the serum metabolomes of mothers and pups (Fig. 1). The mothers clustered in a single group under this scaling whereas the pups separated according to day of lactation, indicative of a marked progression in their metabolomes with time.

We first provide general descriptions of pups’ and mothers’ serum metabolomes and how they change, then deal with the extent to which the sera of pups and mothers progressively differ.

### Serum metabolome of seal pups

**Figure 2:**
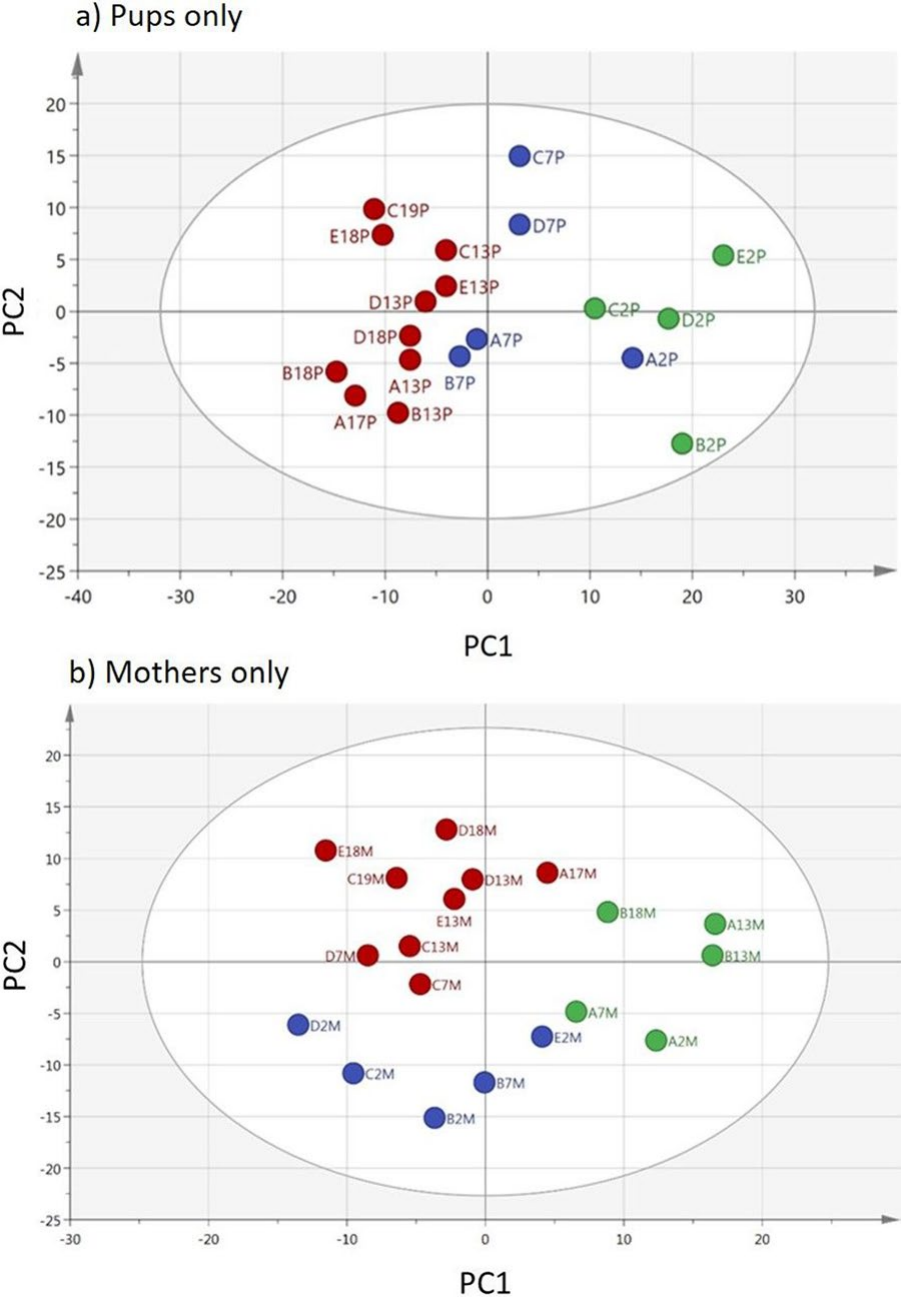
The serum metabolomes of Atlantic grey seal pups with time after birth. (**a**) PCA analysis of pup sera based on 615 features in positive and negative ion mode. Hierarchical cluster analysis groups the 13–19 day samples into one group (red) but separates, with one exception, the 2 and 7 day samples (green and blue). One sample was excluded from the analysis since it lay outside of the ellipse. (**b**) PCA analysis of the metabolome of sera from Atlantic grey seal mothers with time after birth based on the same compounds as for pups in positive and negative ion mode. Hierarchical cluster analysis groups the 13–19 day samples into one group (red) but separates, with one exception, the 2 and 7 day samples (blue and green, respectively). Any sample that lay outside of the ellipse was excluded from the analysis. Data point codes – the initial letter is the seal mother and pup pair (**A to E**), the numeral is the days after birth, then ‘M’ for mother.

The serum metabolome of pups diverges dramatically and continuously over time from the first to the latest sampling times (Fig. 2A). Also evident is a considerable diversity among individual pups. While PCA analysis gives a clear separation according to day of lactation (discussed below), the heat map shown in Supplementary Fig. S2 suggests that the serum metabolome of the pups is fairly stable for the most abundant features, particularly over the early days. The most highly abundant metabolites include lysophosphatidyl choline (LPC) lipids, which are important intermediates in cell membrane biosynthesis but also in distributing polyunsaturated lipids to sites such as the brain^20–22^. LPCs are believed to be produced by the liver and exported to extra-hepatic tissues bound to albumin in the plasma^21^. They are highly abundant in the serum, and there appears to be a change in bias from LPC16:0 to LPC 18:0 with time, perhaps reflecting the biosynthesis of longer chain PC lipids for incorporation into cell membranes. Proline is particularly important for the biosynthesis of collagen, requiring its hydroxylated form, and is also stably abundant in pup serum over time. The essential amino acids leucine and valine are plentiful, consistent over time, and are likely important for the high levels of protein synthesis in rapidly growing tissues such as muscles.

A statistical analysis of pup serum metabolomes between days after birth using ANOVA with Tukey’s HSD test is given in Supplementary Table S1. This showed significant metabolic changes in the pups with 140 metabolites varying significantly between two or more days, and particularly marked variations in pyrimidine and purine metabolites, in a number of amino acids, in tryptophan metabolism and in lipid metabolism.

### Pups – serum metabolites that decline with time

**Figure 3:**
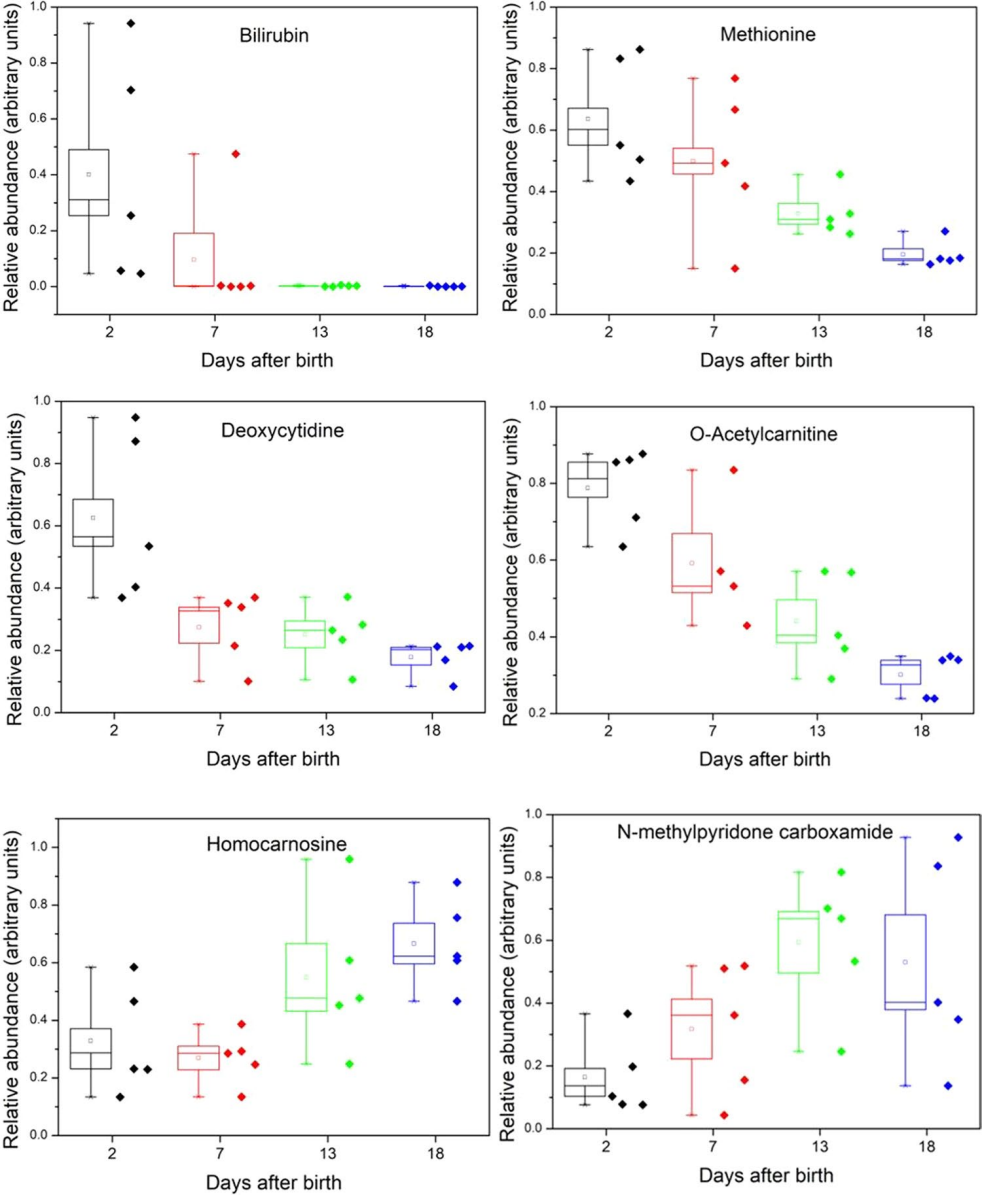
Changes in selected metabolites in grey seal pup serum with time after birth. Box and whisker settings - line in box, median; square in box, mean; box boundaries, standard error; whisker bars, range. The scatter points refer to values for individual pups. The days after birth labelled 18 are a combination of samples taken at 17, 18, or 19 days. Lactations periods lasted 18 to 21 days. For all the metabolites significant differences exist between the population means by ANOVA with Tukey’s test at the 0.05 level or below. Supplementary Table S1 provides statistical analysis of where significant differences were found between the days for these and other metabolites.

Closer analysis of the effect of day after birth on the serum metabolome of the seal pups was carried out by using CV-ANOVA to support the strength of the model, and orthogonal partial least squares analysis (OPLS) to predict the day of sampling based on key metabolites. A strong model was obtained based on six metabolites (CVANOVA score 1.4 × 10^−7^; Supplementary Figs. S3A,B) in which bilirubin stands out. Bilirubin is associated particularly with day 2 post-parturition samples, and essentially vanishes thereafter (Fig. 3). Bilirubin is a breakdown product of haem metabolism in the liver, has been observed in the blood of newborn harbour seals^23^, and is commonly found in pregnant and neonatal Carnivora^24^ and humans^25,26^. In the context of gestation and birth, serum bilirubin in neonates rises as a result of transplacental or colostrum-derived maternal antibodies causing haemolytic disease, or due to trauma during birth - erythrolysis from these mechanisms liberates haemoglobin, which in turn is processed to release bilirubin into circulation^25,26^. Circulating bilirubin may be removed from the pups as the liver and intestines become more functionally mature, by excretion into bile, or degradation by exposure of skin to light^25,26^. The large differences in amounts of bilirubin among day 2-sampled pups could be due to the extent of haemolysis differing between individual pups, and/or differences in the exact timings of sample collection after birth. The placentae of Carnivora have a circumferential haematophagous zone that is a site of erythrocyte breakdown^27,28^, and this may also supply bilirubin to the foetus before or during birth. Curiously, the rapid collapse in bilirubin in the pups after birth contrasts with its rise in the mothers (Supplementary Fig. S4).

An alternative mechanism for the appearance of bilirubin is erythrocyte turnover in the changeover from foetal to adult haemoglobin^29^, though the process whereby foetal haemoglobin-containing erythrocytes are removed from circulation (such as by sequestration by the liver or spleen) might not involve release of haem into circulation, and the changeover is likely to be more gradual than observed here.

Several metabolites that are strongly associated with metabolism and development decline over time in pups. Methionine shows a marked fall between day 2 and day 18 after birth in pups and, as an essential amino acid, may be a limiting nutrient (Fig. 3). Other than in protein synthesis, methionine is essential for many cellular processes including the production of nucleobases, and reactions such as methylation of DNA and histone proteins^30^. In comparison, other essential amino acids such as phenylalanine and tryptophan are fairly stable in pups (Supplementary Fig. S5).

Overall, uridine declines slightly with time, albeit with substantial variability between pups whose individual levels remain fairly consistent (Supplementary Fig. S5). Deoxycytidine, on the other hand (Fig. 3), undergoes a notable decline, suggesting that there may be a limiting supply of glucose into the pentose phosphate pathway, glucose being required for the production of the ribose and deoxyribose necessary for nucleotide synthesis.

Acetyl carnitine (Fig. 3) declines steadily and markedly over time (as it also did in grey seal milk (Supplementary Fig. S6; ref. ^18^), and in pup sera it may be the result of a decrease in supply via milk with L-carnitine. Alternatively, the decline may indicate that energy supplies are low, since acetyl carnitine is derived from β-oxidation of fatty acids and can be transferred to the tricarboxylic acid (TCA) cycle. It may be that it can be produced in one part of the body and exported to other tissues such as muscle to support energy metabolism. The contemporaneous decline of acetyl carnitine in pup serum (Fig. 3) with its decline in milk (Supplementary Fig. S6; ref. ^18^) suggests that it is an important maternally-derived nutrient that may become limiting. Interestingly, elephant seal pups have been found to defend their total plasma carnitine levels into their protracted post-weaning fast, whereupon they maintain acylcarnitine levels while the total carnitine levels decrease with time after weaning^31^.

The decline of another abundant metabolite, guanidino butanoate, is similarly puzzling. This is a major metabolite of the neurotransmitter gamma-aminobutyric acid (GABA)^32^, and presumably important in the development of functioning of neural tissues.

### Pups – serum metabolites that increase with time

A few metabolites increase with time in pups, such as homocarnosine and N-methyl pyridone carboxamide (Fig. 3). The significance of the increase in homocarnosine is not entirely clear, since it is a metabolite of GABA and is generally confined to the brain^33^. Of more immediate interest because of a potential association with starvation, is the increase exhibited by N-methyl pyridone carboxamide (Fig. 3), which is a marker of peroxisome proliferation (^34^). An increase for this compound was also observed in the milk of the mothers investigated here (Supplementary Fig. S6; ref. ^18^), possibly a result of a reduction of glomerular filtration rate in a mother as fasting progresses during lactation. Its pattern in the pups’ sera could therefore be due as much to transfer in milk as to the pups’ intrinsic metabolisms. Puzzlingly, however, the renal filtration rate in lactating elephant seals has been observed to increase^35^.

### Maternal serum metabolomes

Excluding the considerably more diverse pup samples from the HCA/PCA analysis shown in Fig. 1 reveals that maternal serum metabolomes are remarkably stable, further emphasised in the heat map shown in Supplementary Fig. S7, but can be separated according to day of lactation (Fig. 2B). We did not find a single metabolite signal that may indicate deterioration in maternal metabolisms with time, but there is a strong fit to the OPLSDA model (CVANOVA score = 5.4 × 10^−7)^ according to day based on six compounds - leucine, adenosine, nicotinamide, sulfocatachol, N-(hexadecanoyl)-sphing-4-enine-1-phosphocholine (SP16:0), and eicosanoic acid (Supplementary Figs. S8A,B) - of these, the essential amino acid leucine, and adenosine fall steadily with time. Adenosine is demanding of nitrogen availability for its synthesis, as well as glucose to provide ribose. It may therefore be that a combination of metabolites, rather than a single one, will provide an indication of maternal metabolic status. The statistical analysis given in Supplementary Table 1 again indicates that the mothers’ metabolomes were relatively stable with only 41 metabolites varying between two or more days compared to 140 metabolites in the pups. Perhaps significantly according to the ANOVA analysis of the mother’s metabolome, there were highly significant variations in three carnitines indicating some variation in fat metabolism over the time period.

### Comparison between mother and pup serum metabolomes

The most abundant metabolites in the pups and mothers are at similar levels, with LPC 18:0, LPC 16:0, LPC 18:1, guanidino butyrate, creatine, creatinine and proline all in relative abundance (Supplementary Figs. S2 and S7). Carnitine is abundant in mothers and pups, being slightly more so in the mothers. The most abundant metabolites are generally stable throughout the sample period in the mothers, though there are fewer lysolipids than in pups, perhaps suggesting less active biosynthesis of new cell membranes compared to rapidly growing pups. Significantly, lysolipids are also important as signalling molecules in a wide range of biological process such as cell division, angiogenesis, inflammation, and both innate and adaptive immune systems^36–40^. The essential amino acids leucine, phenylalanine, tryptophan, and also taurine are more abundant in pups’ than mothers’ sera, particularly in the case of leucine of which the mothers’ sera appear to become slightly depleted with time, which is also the case with taurine. Some hypercarnivores (as are seals) are unable to synthesise taurine, so are entirely dependent on dietary sources^41,42^ - whether this is true of seals is unknown and merits attention.

**Figure 4:**
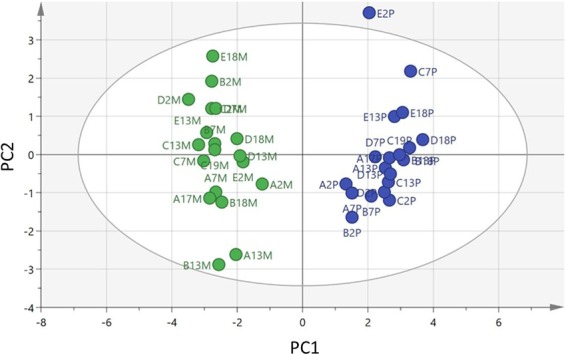
OPLSDA separation of mothers and pups based on four key metabolites, L-citrulline, tyrosine, N6-methyl-L-lysine, and L-arginine (CV-ANOVA 5.2 × 10^−19^). See also Table 1. Green – mothers. Blue – pups.

Overall, the more abundant metabolites do not strongly differentiate the serum metabolomes of mothers and pups in the PCA model. But the separation between the two can be reduced to a strong OPLSDA model (CVANOVA 5.2 × 10^−19^) when based on four core metabolites, namely N-methyllysine, arginine, citrulline, and tyrosine (Fig. 4 and Table 1). These may provide a potentially useful set of markers for following metabolic changes leading up to weaning. The levels of arginine and citrulline are much higher in sera from the pups in comparison with the mothers (Table 1). Citrulline is the product resulting from the formation of nitric oxide from arginine^43^ and its relative abundance suggests a potential importance of nitric oxide in promoting processes such as angiogenesis in the developing pups^43^. The roles of the other two markers, tyrosine and N6-methyl-L-lysine, are not immediately apparent, although the former is classed as a conditionally essential amino acid and is synthesised by mammals from a true essential amino acid, phenylalanine.

**Table 1:** Metabolic discrimination of mothers and pups.

Metabolite	P value	Ratio pups/mothers
L-Citrulline	1.13E-18	20.62
Tyrosine	1.63E-14	11.55
N6-Methyl-L-lysine	1.71E-09	36.69
L-Arginine	1.73E-09	10.04

Ratio of four key metabolites in 19 samples from mothers in comparison with 19 samples from pups taken over the sample period. See Fig. 6 for the associated PCA plot.

### Metabolite stockpiling by pups

**Figure 5:**
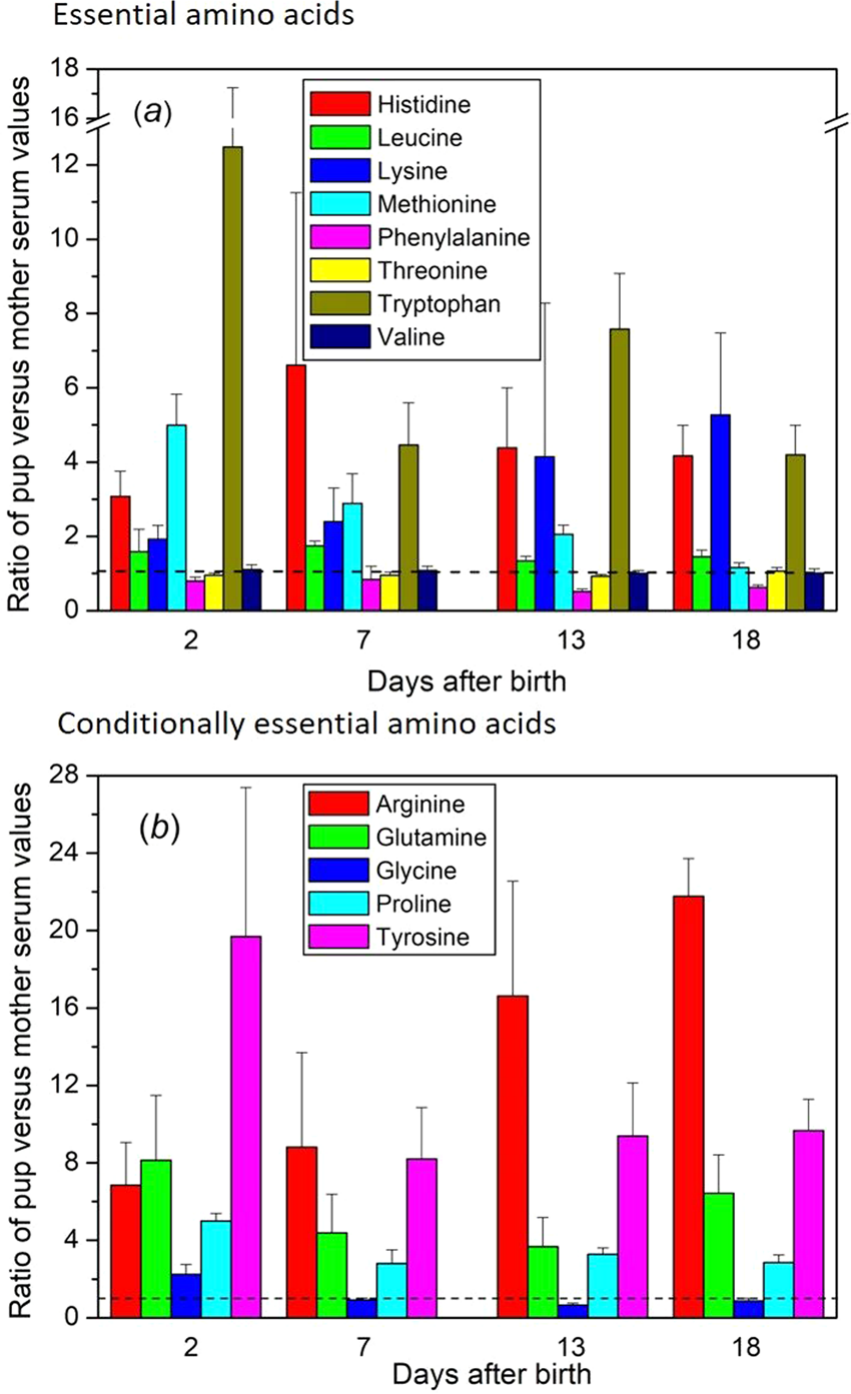
Selective overabundance of essential (**a**) and ‘conditionally essential’ (**b**) amino acids in pup serum relative to maternal serum. Relative abundance in the sera of pups with time after birth expressed as a ratio with the concentration in their individual mothers’ sera (ratio of pup/mother; mean + SE). Conditionally essential amino acids are classed as such because their synthesis can be limited under certain pathophysiological conditions, such as prematurity in an infant or in individuals in severe catabolic distress. The days after birth labelled 18 are a combination of samples taken at 17, 18, or 19 days. The horizontal dotted lines indicate equivalence between levels in mothers and pups. Isoleucine (essential) and cysteine (conditionally essential) are not included because they are not resolved in our analytical system. Some amino acids occurred in modified form, such as N6-Methyl-L-lysine, which had overabundance levels in pup:mother ratios of 37.7, 41.5, 84.8, and 83.3 on days 2, 7, 13, and 18, respectively (for clarity, not included in graph – see Supplementary Figure S9). Only threonine, and valine did not show differences between days by ANOVA with Tukey’s test at the 0.05 level or below, or by two-sample t-test between days for deviation from unity. More detailed box and whisker plots with individual ratio values of the data are given in Supplementary Figure S9. Supplementary Table S1 provides statistical analysis of where significant differences were found between the days for each of the compounds.

**Figure 6:**
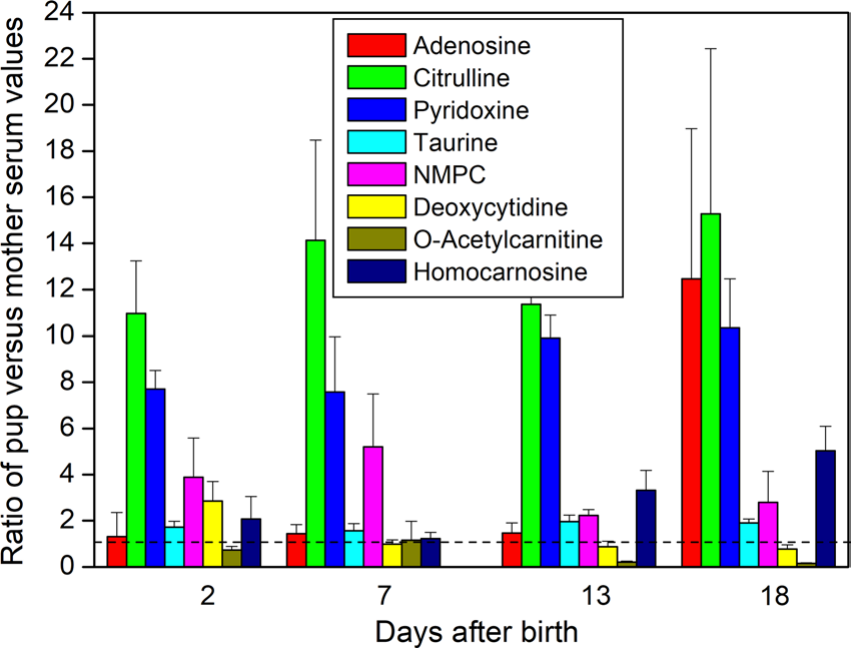
Metabolites that are enriched in pups’ sera relative to that of their respective mothers. Citrulline is a precursor to arginine, an intermediate in the urea cycle, and a by-product in the enzymatic production of nitric oxide from arginine by nitric oxide synthase. Pyridoxine (Vitamin B_6_) is required for synthesis of amino acids, carbohydrates, lipids, and haemoglobin, for which it is also involved in modifying haemoglobin’s affinity for oxygen. Adenosine is a component of nucleic acids and intermediates in energy metabolism such as ATP, but is also a neuromodulator and active in vasodilation. NMPC, N1-Methyl-2-pyridone-5-carboxamide. Detailed box and whisker plots of the data also showing individual ratio values are given in Supplementary Figure S10. See Supplementary Table S1 for full statistical analysis of where significant differences were found between the days for each of the compounds. See legend for Fig. 5 for details of how the data are calculated and presented.

The starting points of this work were to examine the degree to which mother seals may sacrifice their own metabolic security during their short, intense lactations, and also how the brief nursing period of pups prepares them for a post-weaning month on land during which they undergo further development without nourishment. One expectation for the latter question may be that pups accumulate essential nutrients to levels that are proportionately greater than in the mothers. Examples of where this applies are given in Figs. 5 and 6. Several essential amino acids are hugely elevated in the pups compared to the mothers, and others hover around equivalence (Fig. 5a). The only source for these nutrients is milk, and ultimately the mother’s diet and body reserves before she comes to land.

Most of the amino acids that are classified as being essential only under certain stressed physiological conditions (‘conditionally essential’) are also seen to accumulate in pups (Fig. 5b). Notable among these is arginine, which is conditionally essential in that immature and rapidly growing individuals may require arginine in their diets^44^. For some carnivores, such as dogs, cats, and ferrets, dietary arginine is deemed to be essential because their protein catabolism generates large quantities of ammonia that needs to be processed through the urea cycle (for dogs see ref. ^45^), and if insufficient arginine is available for supply of ornithine to the urea cycle, the resulting ammonia can become toxic^46^. For regular meat eaters, however, this may not be a difficulty because meat contains sufficient arginine. Whether this reliance on exogenous arginine applies to seals, being hypercarnivorous, is not known. However, for an immature animal, despite consuming milk rather than meat, there may still be a need for arginine. Also of note, therefore, is the dramatic relative accumulation of citrulline in pups, which, as noted above, is a precursor to arginine in the arginine biosynthetic pathway (Fig. 6). Citrulline is also a product of nitric oxide production from arginine, nitric oxide being crucial to a range of processes that are likely to be particularly important to a neonate, such as the immune system, vasodilation, and angiogenesis^43,47^.

Amino acids that the pups should be able to synthesise themselves (“non-essential”) also rise considerably in pups above maternal levels (Supplementary Fig. S9) during the lactation period. These accumulate from maternal donation, which would obviate subsequent expenditure of energy and resources in their synthesis by pups.

Other classes of metabolites occur at many times higher levels in pups than mothers (Fig. 6) including one, pyridoxine (Vitamin B_6_), the only source of which must be the mother’s diet. This acts as a cofactor in a wide range of core processes such as in the synthesis of haem for placement into haemoglobin^48,49^, and in the functioning of haemoglobin itself in modifying its affinity for oxygen^50,51^. High levels of haemoglobin and haem-containing myoglobin in particular are crucial for a diving mammal such as grey seals^52^. Other metabolites that appear at dramatically high relative levels in pups include adenosine, levels of which are particularly high shortly before the mothers depart (Fig. 6). Adenosine has many roles in addition being important for ATP biosynthesis and for the biosynthesis of Co-enzyme A which is required for fatty acid metabolism. In addition, it is important as a neuromodulator, particularly in the brain. The levels of adenosine in two of the mothers were very high on day 2 of lactation but then fell rapidly to the low levels present in the other mothers then and later (data not shown). If this were indicative of general high levels of adenosine in the brain immediately after birth then it could be reflective of profound periparturient behavioural changes^53^.

It must be noted that these ratios represent relative proportions of compounds between mother and pup sera, and do not relate to absolute concentrations in serum or tissues. Overall, though, the mothers, via their milk, are enabling their pups to accumulate crucial metabolites to considerably higher levels than in their own blood. So, while the pups appear to be stockpiling, the degree to which this is dangerously depleting the mothers’ reserves remains to be seen. Moreover, we do not know whether the mothers’ metabolomes differ between lactation and non-lactation, or before they emerge from sea to give birth. Nevertheless, our findings provide insight into the extraordinary nutritional relationship between mother and pup in grey seals.

## Conclusions

Despite the many changes observed in both mother and pup serum metabolomes, we found a remarkable degree of metabolic stability overlaying notable changes in certain indicator compounds. The pups exhibited the greatest degree of change and clear progression with time, which is not surprising given their move from an *in utero* existence to the end of nutritional support in less than twenty days. Meanwhile, a fall in a potential indicator of mitochondrial metabolic activity (O-acetylcarnitine) was observed in pups that may indicate a temporal reduction in the adequacy of pups’ energy supplies for continued rapid growth and development. Parallel increases in a potential indicator of stress in the fat metabolism of pups (N-methylpyridone carboxamide) may also indicate the onset of limiting nutrition. Intriguingly, there are strong indications that the mothers sacrifice themselves in assisting their offspring to accumulate certain compounds that they, both pups and mothers, cannot synthesise themselves, or that may spare the pup later resource expenditure in having to synthesise them. This particularly includes amino acids, and at least one essential vitamin (B_6_) involved in a wide range of metabolic processes.

One of the original aims of this work was to seek indicators of whether a mother had reached the end of her nutritional tolerance for staying on land and lactating and was about to leave. This would, for example, be useful to monitor whether maternal food resources for raising pups adequately may be altering with climate changes or human-mediated changes in food resources, with consequent implications for population stability or recovery. We did not find a strong single metabolite factor indicative of approaching maternal exhaustion, but instead a set of key metabolites (leucine, adenosine, nicotinamide, sulfocatachol, N-(hexadecanoyl)-sphing-4-enine-1-phosphocholine, and eicosanoic acid) that may together be useful markers or proxies for progressive changes in maternal metabolism throughout lactation. Whether these may be used to indicate when a mother may terminate lactation and go to sea remains to be seen, and whether the changes in them cause a mother to leave is speculative, but they are clearly worthy of investigation in grey seals and other phocids that undergo lactation fasts. They may also be useful in detecting body resource perturbations in mother seals under adverse conditions such as limitations in food supplies before they come to land to give birth, with consequent effects on how they endure lactation. Similarly, the factors observed to accumulate in pups may be similarly useful in gauging adequacy of nutrition before weaning, and thus as indicators of chance at survival. In this context, it would be intriguing now to follow what happens to the pups’ metabolisms between the cessation of milk nutrition and their departure to sea, which can be as much as 40 days later.

## Materials and Methods

### Ethical statement

Work involving animals in this study was licensed under UK Home Office project 60/4009 or preceding versions and conformed to the UK Animals (Scientific Procedures) Act, 1986. Research was approved by the University of St Andrews Animal Welfare and Ethics Committee.

### Serum samples

Blood samples were collected from 21 mother-pup pairs on the Isle of May, Scotland in 2015. Five of these (pairs coded A to E) provided samples on four occasions during the average 18 days between birth and just before the mothers were expected to wean and return to sea. Regular surveys of the colony identified known animals and their pupping dates. To avoid desertions by the mothers, the earliest samplings were during day 2 post-partum, with subsequent samplings on days 7 and 13, followed by days 17, 18, or 19 (for ease of presentation, samples taken 17,18, or 19 days after birth are presented as day 18 in graphs). Females were tranquilised with a mass-adjusted dose of Zoletil 100 (Virbac, Bury St Edmunds, Suffolk, UK), followed by intravenous oxytocin to stimulate milk let-down to provide milk samples, and given an intramuscular prophylactic dose of tetracycline, as reported previously^18^. Blood was collected into vacutainers, allowed to clot and serum was separated by centrifugation within 4 hours of collection, frozen at −20 °C and stored at −20 °C or below until sent for metabolomic analysis.

### Chemicals and solvents

HPLC grade acetonitrile (ACN), propanan-2-ol, and water were purchased from Fisher Scientific, UK. Ammonium carbonate was purchased from Sigma-Aldrich (Poole, UK). Authentic stock standards were prepared as described previously^54^ and diluted four times with ACN before LC-MS analysis, then distributed into seven different standard solutions.

### Sample treatment

Samples were stored and kept frozen at −20 °C until transfer to the metabolomics laboratory where they were stored at −80 °C until analysis. Aliquots of thawed serum samples (0.2 ml) were transferred to Eppendorf tubes and were then vortex mixed with 0.8 ml of ACN. The samples were centrifuged at 3000 g for 10 min. and the supernatants transferred to HPLC vials.

### LC-MS analysis

LC-MS analysis was carried out similarly to previous reports^18,55^, briefly as follows. An Accela HPLC system interfaced to an Exactive Orbitrap mass spectrometer (Thermo Fisher Scientific, Bremen, Germany) was used for the liquid chromatographic separations on a ZIC-pHILIC (150 × 4.6 mm, 5 µm) HPLC column supplied by HiChrom (Reading, UK). Samples were run on LC-MS under the following conditions: the ZIC-pHILIC mobile phase consisted of 20 mM ammonium carbonate in HPLC-grade water (A) and acetonitrile (B); the solvent gradient was 80% B (0 min), 20% (30 min), 8% (31–36 min), and 80% (37–45 min) at a flow rate of 0.3 mL/min. The nitrogen sheath and auxiliary gas flow rates were maintained at 50 and 17 arbitrary units. The electrospray ionisation (ESI) interface was employed in a positive/negative dual polarity mode, with a spray voltage of 4.5 kV for positive mode and 4.0 kV for negative mode, while the ion transfer capillary temperature was set at 275 °C. Full scan data were obtained in the mass-to-charge ratio (m/z) between 75 and 1200 amu for both ionisation modes. The data were collected and processed using Xcalibur 2.1.0 software (Thermo Fisher Scientific, Bremen, Germany).

### Data processing

Raw LC-MS files were processed as previously^18,55^ and briefly as follows by using m/z Mine 2.14 and the accurate masses were searched against an in house metabolite database prepared by including data from the Human Metabolome database, Lipid Maps and the Metlin database. Graphical representations, tabular features and statistical analysis (p-value generation) were performed in Excel (Microsoft Office 2013). SIMCA-P version 14.1 (Umetrics, Sweden) was used for multivariate analysis which included PCA, OPLS-DA and Orthogonal Partial Least Squares (OPLS). The data were centred, and Pareto scaled for PCA, OPLS-DA and OPLS. The data were also loaded into Metaboanalyst 4^56^ and subjected to ANOVA with Tukey’s HSD test.

All of the metabolomics data used for graphs, tables, and statistical analyses are available in online data supplement entitled “Grey seal mother and pup serum metabolome data”.

## Supplementary information

Supplementary Information.

Supplementary Information.
