# Supplementary material for: Stockpiling by pups and self-sacrifice by their fasting mothers observed in birth to weaning serum metabolomes of Atlantic grey seals
Source: Sci Rep. 2020 May 4;10:7465. doi: 10.1038/s41598-020-64488-1 (PMC7198541; doi:10.1038/s41598-020-64488-1)
Supplement: Supplementary file 1 — Supplementary Information. [file 41598_2020_64488_MOESM1_ESM.pdf]

# Stockpiling by pups and self-sacrifice by their fasting mothers observed in birth to weaning serum metabolomes of Atlantic grey seals

David G. Watson[1], P.P (Paddy) Pomeroy[2], Naser F. Al-Tannak[1,3], and Malcolm W. Kennedy[4]

[1] Strathclyde Institute of Pharmacy and Biomedical Sciences, University of Strathclyde, 161 Cathedral Street, Glasgow G4 0RE, Scotland, UK. D.G.Watson@strath.ac.uk

[2] Sea Mammal Research Unit, Scottish Oceans Institute, University of St Andrews, St Andrews, Fife, Scotland, United Kingdom. pp6@st-andrews.ac.uk

[3] Department of Pharmaceutical Chemistry, Faculty of Pharmacy, Kuwait University, P.O. Box 23924, Safat, 13110 Kuwait City, Kuwait; dr_altannak@HSC.EDU.KW

[4] Institute of Biodiversity, Animal Health & Comparative Medicine, Graham Kerr Building, College of Medical, Veterinary and Life Sciences, University of Glasgow, Glasgow G12 8QQ, Scotland, UK, malcolm.kennedy@glasgow.ac.uk

Authors for correspondence: Malcolm Kennedy (malcolm.kennedy@glasgow.ac.uk) and David Watson (D.G.Watson@strath.ac.uk)

## Electronic supplementary material

**Figure S1.** Appearance of serum samples of mother and pup Atlantic grey seals at the times indicated after birth.

**Figure S2**. The Top 50 metabolites by abundance in the serum of Atlantic grey seal pups from soon after birth to weaning.

**Table S1.** Statistical analysis of the serum metabolomics profiles of mother and pup grey seals using ANOVA with Tukey's HSD tests.

**Figure S3A.** OPLS plots of pups predicted versus actual day based on six metabolites.

**Figure S3B.** Loadings plot for OPLS plot for pups of observed versus predicted day based on six metabolites.

**Figure S4.** Reciprocal trends in serum levels of bilirubin in pup and mother grey seals with time after birth.

**Figure S5.** Examples of compounds exhibiting relative stability with time, and diversity in levels, between individual pup.

**Figure S6.** Changes in metabolites related to energy metabolism in grey seal milk with time after birth.

**Figure S7.** The top 51 metabolites in serum by abundance from soon after birth to imminent weaning in Atlantic grey seal mothers.

**Figure S8A.** OPLS plots showing strong fit between mothers' samples and day after birth based on six metabolites.

**Figure S8B.** Loadings plot for OPLS plot for mothers of observed versus predicted day.

**Figure S9.** Detailed box and whisker plots of the data presented as bar charts in Figure 5a (essential amino acids) and Figure 5b (conditionally amino acids) of the main text.

**Figure S10.** Detailed box and whisker plots of the data presented on selected compounds as bar charts in Figure 6 of the main text.

**Figure S11.** Selective overabundance of some non-essential amino acids in pup serum relative to mothers.

**References.**

## Abbreviations

amu, atomic mass units; ACN, acetonitrile; CV-ANOVA, cross-validated residuals analysis of variance; GABA, gamma-aminobutyric acid; GC, gas chromatography; HCA, hierarchical cluster analysis; HILIC, hydrophilic interaction liquid chromatography; HRMS, high resolution mass spectrometry; LC, liquid chromatography; LPC, phosphatidyl choline; MA, multivariate analysis; m/z, mass to charge ratio; MS, mass spectrometry; OPLS, orthogonal partial least square regression analysis; OPLS-DA , orthogonal partial least squares discriminant analysis; PC, principal component; PCA, principal components analysis; TCA, tricarboxylic acid cycle/Krebs cycle; VIP, variable importance projection.

**Figure S1.** Appearance of serum samples of mother and pup Atlantic grey seals at the times indicated after birth. The samples had been stored frozen until use here.

**Time after birth (2, 7, 13, and 17 or 18 days)**

**|Mothers →** **|Pups →**

**Seal A**

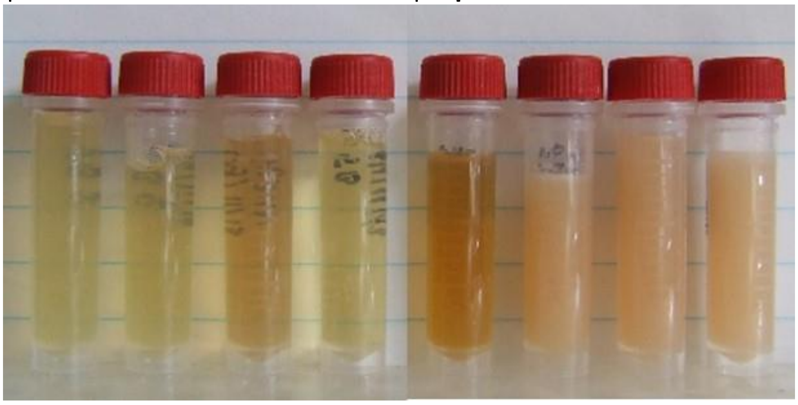

**Seal B**

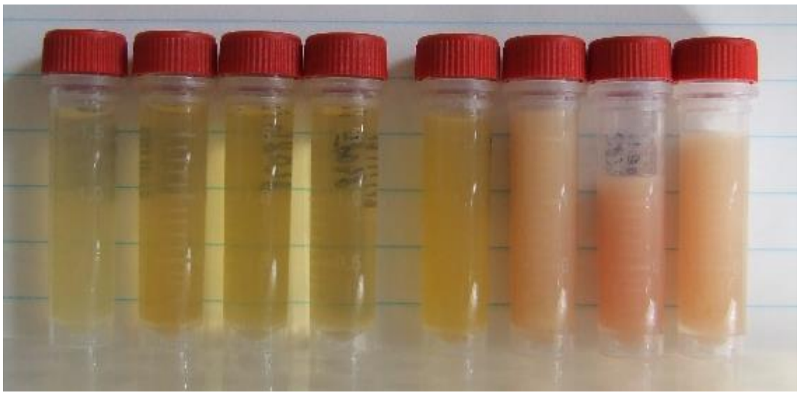

**Seal D**

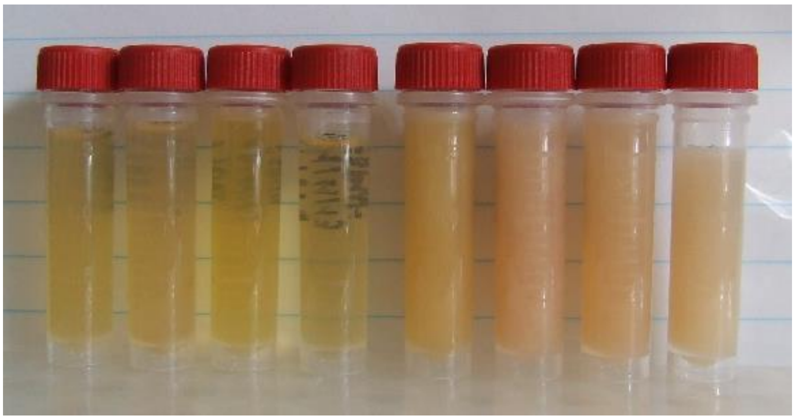

**Figure S2**. The Top 50 metabolites by abundance in the serum of Atlantic grey seal pups from soon after birth to weaning. Column codes - the initial letter is the seal mother/pup pair (A to E), the numeral is the days after birth, then 'P' for pup. Relative abundance/signal magnitude colour codes from red for highest to blue for lowest; red = 1 X $10^9$, yellow = 5 X $10^7$, blue = 2 X $10^7$. M/Z, mass to charge ratio. Rt, column retention time in minutes. The E7P sample laid well outside the ellipse in the PCA model and was an outlier, reason unknown, so is not included here. m/z, mass divided by charge number. Rt, retention times on the ZICpHILIC columns.

## Pups

| M/Z | Rt(min) | **Chemical Formula** | **Compound** | A2P | B2P | C2P | D2P | E2P | A7P | B7P | C7P | D7P | A13P | B13P | C13P | D13P | E13P | A17P | B18P | C19P | D18P | E18P |
|---|---|---|---|---|---|---|---|---|---|---|---|---|---|---|---|---|---|---|---|---|---|---|
| 496.340 | 4.8 | C24H50NO7P | LPC16:0 | | | | | | | | | | | | | | | | | | | |
| 146.092 | 14.9 | C5H11N3O2 | Guanidino butanoate | | | | | | | | | | | | | | | | | | | |
| 522.355 | 4.7 | C26H52NO7P | LPC18:1 | | | | | | | | | | | | | | | | | | | |
| 524.371 | 4.6 | C26H54NO7P | LPC18:0 | | | | | | | | | | | | | | | | | | | |
| 116.071 | 12.6 | C5H9NO2 | L-Proline | | | | | | | | | | | | | | | | | | | |
| 132.077 | 14.4 | C4H9N3O2 | Creatine | | | | | | | | | | | | | | | | | | | |
| 114.066 | 9.6 | C4H7N3O | Creatinine | | | | | | | | | | | | | | | | | | | |
| 544.340 | 4.7 | C28H50NO7P | LPC20:4 | | | | | | | | | | | | | | | | | | | |
| 550.387 | 4.6 | C28H56NO7P | LPC20:1 | | | | | | | | | | | | | | | | | | | |
| 435.252 | 4.8 | C21H41O7P | LPA18:1 | | | | | | | | | | | | | | | | | | | |
| 468.308 | 5.0 | C22H46NO7P | LPC14:0 | | | | | | | | | | | | | | | | | | | |
| 409.236 | 4.8 | C19H39O7P | LGP16:0 | | | | | | | | | | | | | | | | | | | |
| 782.570 | 4.1 | C44H81NO8P | PC36:4 | | | | | | | | | | | | | | | | | | | |
| 162.112 | 13.1 | C7H15NO3 | L-Carnitine | | | | | | | | | | | | | | | | | | | |
| 760.586 | 4.1 | C42H82NO8P | PC34:1 | | | | | | | | | | | | | | | | | | | |

| | | | |
|---|---|---|---|
| 808.585 | 4.0 | C46H82NO8P | PC38:5 |
| 810.601 | 4.0 | C46H84NO8P | PC38:4 |
| 258.110 | 14.2 | C8H20NO6P | GPC |
| 542.324 | 4.7 | C28H48NO7P | LPC20:5 |
| 132.102 | 10.7 | C6H13NO2 | L-Leucine |
| 118.086 | 12.4 | C5H11NO2 | L-Valine |
| 309.280 | 3.8 | C20H38O2 | eicosenoic acid |
| 520.340 | 4.8 | C26H50NO7P | LPC18:2 |
| 780.554 | 4.1 | C44H78NO8P | PC36;5 |
| 205.097 | 11.5 | C11H12N2O2 | L-Tryptophan |
| 301.218 | 3.8 | C20H30O2 | eiccoapentaenoic acid |
| 127.050 | 11.3 | C5H6N2O2 | Thymine |
| 437.268 | 4.6 | C21H43O7P | LGP18:0 |
| 303.233 | 3.8 | C20H32O2 | eicosatetraenoic acid |
| 166.086 | 10.0 | C9H11NO2 | L-Phenylalanine |
| 806.570 | 4.1 | C46H80NO8P | PC38:6 |
| 758.570 | 4.1 | C42H80NO8P | PC34:2 |
| 128.071 | 10.3 | C6H9NO2 | Tetrahydropyridine-2-carboxylate |
| 786.601 | 4.1 | C44H84NO8P | PC36:2 |
| 510.356 | 4.7 | C25H52NO7P | LPE20:0 |
| 204.123 | 10.9 | C9H18NO4 | O-Acetylcarnitine |
| 337.312 | 3.7 | C22H42O2 | docosenoic acid |
| 124.008 | 14.4 | C2H7NO3S | Taurine |
| 90.055 | 14.5 | C3H7NO2 | L-Alanine |
| 182.081 | 12.8 | C9H11NO3 | L-Tyrosine |
| 482.324 | 4.9 | C23H48NO7P | LPE16:0 |
| 732.554 | 4.1 | C40H78NO8P | PC32:1 |
| 279.233 | 3.8 | C18H32O2 | Linoleate |

| | | | |
|---|---|---|---|
| 156.077 | 15.0 | C6H9N3O2 | L-Histidine |
| 788.617 | 4.1 | C44H86NO8P | PC36:1 |
| 568.340 | 4.6 | C30H50NO7P | LPC22:6 |
| 508.340 | 4.8 | C25H50NO7P | LPE20:0 |
| 170.092 | 12.7 | C7H11N3O2 | Methyl-L-histidine |
| 104.071 | 12.0 | C4H9NO2 | 4-Aminobutanoate |
| 329.249 | 3.8 | C22H34O2 | docosatetraenoic acid |

**Table S1.** Statistical analysis of the serum metabolomics profiles of mother and pup grey seals using ANOVA with Tukey's HSD tests. As observed from the multivariate modelling detailed in the main text, there were significant metabolic changes in the pups with 140 metabolites varying significantly between two or more days. There were particularly marked variations in pyrimidine and purine metabolites, in a number of amino acids, in tryptophan metabolism and in lipid metabolism. In contrast the mothers' metabolomes were stable with only 41 metabolites varying between two or more days. Perhaps significantly according to the ANOVA analysis of the mother's metabolomes there were highly significant variations in three carnitines indicating some variation in fat metabolism over the time period. Data analysed using Metaboanalyst 4 [1].

**Pups**

ANOVA with Tukey's test and FDR values indicating metabolites which change significantly between the days.

| RT in | MW | Metaboloite | f.value | p.value | FDR | Tukey's HSD |
|---|---|---|---|---|---|---|
| **Purine and Pyrimidine Biosynthesis** | | | | | | |
| 15.4 | 75.0320 | Glycine | 7.1 | 2.9E-03 | 1.8E-02 | Day2-Day13; Day2-Day18; Day7-Day2 |
| 11.5 | 112.0273 | Uracil | 20.3 | 1.1E-05 | 2.8E-04 | Day2-Day13; Day7-Day13; Day2-Day18; Day7-Day18 |
| 14.4 | 114.0429 | 5,6-Dihydrouracil | 14.7 | 7.4E-05 | 1.1E-03 | Day2-Day13; Day2-Day18; Day7-Day18 |
| 10.7 | 125.0589 | 5-Methylcytosine* | 21.7 | 7.0E-06 | 2.2E-04 | Day2-Day13; Day7-Day13; Day2-Day18; Day7-Day18 |
| 9.4 | 125.0589 | 5-Methylcytosine | 6.4 | 4.5E-03 | 2.5E-02 | Day2-Day13; Day7-Day2 |
| 8.3 | 126.0429 | Thymine | 6.7 | 3.8E-03 | 2.1E-02 | Day2-Day18; Day7-Day18 |
| 13.3 | 128.0586 | 5,6-Dihydrothymine | 9.6 | 7.4E-04 | 6.7E-03 | Day2-Day18; Day7-Day18 |
| 12.3 | 142.0378 | 5-Hydroxymethyluracil¥ | 10.3 | 5.2E-04 | 5.2E-03 | Day2-Day13; Day2-Day18; Day7-Day18 |
| 8.5 | 150.0542 | 1-Methylhypoxanthine | 16.3 | 4.0E-05 | 7.4E-04 | Day2-Day13; Day2-Day18; Day7-Day2 |
| 9.7 | 165.0651 | 3-Methylguanine | 5.6 | 8.1E-03 | 3.8E-02 | Day2-Day18 |
| 10.3 | 227.0906 | Deoxycytidine | 6.4 | 4.8E-03 | 2.6E-02 | Day2-Day13; Day2-Day18; Day7-Day2 |
| 9.3 | 241.1063 | Methyl-deoxycytidine | 6.7 | 3.9E-03 | 2.2E-02 | Day2-Day13; Day7-Day2 |
| 8.5 | 282.0964 | methylinosine | 5.4 | 9.1E-03 | 4.1E-02 | Day2-Day18; Day7-Day2 |
| 11.3 | 257.1012 | 5-Methylcytidine | 20.4 | 1.0E-05 | 2.8E-04 | Day2-Day13; Day2-Day18; Day7-Day2 |
| 10.6 | 295.0569 | Aminoimidazole ribotide* | 18.9 | 1.6E-05 | 4.0E-04 | Day2-Day13; Day7-Day13; Day2-Day18; Day7-Day18 |
| 11.3 | 295.0569 | Aminoimidazole ribotide | 7.1 | 3.0E-03 | 1.8E-02 | Day2-Day13; Day7-Day2 |
| **Amino acids and metabolites** | | | | | | |

| | | | | | | |
|---|---|---|---|---|---|---|
| 14.5 | 89.0477 | L-Alanine | 8.1 | 1.7E-03 | 1.2E-02 | Day2-Day18; Day7-Day18 |
| 15.0 | 103.0633 | 4-Aminobutanoate | 5.2 | 1.1E-02 | 4.6E-02 | Day7-Day18 |
| 11.7 | 101.0477 | 1-Aminocyclopropane-1-carboxylate | 30.7 | 7.1E-07 | 3.1E-05 | Day2-Day13; Day2-Day18; Day7-Day18; Day7-Day2 |
| 11.4 | 113.0477 | (S)-1-Pyrroline-5-carboxylate | 18.7 | 1.8E-05 | 4.1E-04 | Day2-Day13; Day2-Day18; Day7-Day2 |
| 7.6 | 115.0633 | Proline isomer | 7.1 | 3.0E-03 | 1.8E-02 | Day2-Day18; Day7-Day2 |
| 10.7 | 117.0790 | L-Valine | 12.8 | 1.6E-04 | 2.0E-03 | Day2-Day13; Day7-Day18; Day7-Day2 |
| 14.4 | 125.0147 | Taurine | 7.5 | 2.4E-03 | 1.6E-02 | Day2-Day18; Day7-Day2 |
| 14.2 | 131.0582 | hydroxyproline | 15.6 | 5.1E-05 | 8.8E-04 | Day2-Day13; Day2-Day18; Day7-Day18 |
| 12.3 | 129.0790 | L-Pipecolate | 8.2 | 1.6E-03 | 1.2E-02 | Day7-Day13; Day2-Day18; Day7-Day18 |
| 10.9 | 129.0790 | L-Pipecolate | 7.3 | 2.7E-03 | 1.7E-02 | Day2-Day13; Day2-Day18 |
| 10.3 | 127.0633 | 2,3,4,5-Tetrahydropyridine-2-carboxylate | 62.4 | 4.7E-09 | 5.5E-07 | Day18-Day13; Day2-Day13; Day7-Day13; Day2-Day18; Day7-Day18 |
| 8.2 | 127.0633 | 2,3,4,5-Tetrahydropyridine-2-carboxylate | 6.8 | 3.6E-03 | 2.1E-02 | Day2-Day18; Day7-Day18 |
| 11.9 | 131.0946 | L-Leucine | 6.9 | 3.4E-03 | 2.0E-02 | Day2-Day13; Day2-Day18 |
| 12.8 | 131.0946 | isoleucine | 37.9 | 1.7E-07 | 8.4E-06 | Day2-Day13; Day2-Day18; Day7-Day18; Day7-Day2 |
| 10.1 | 140.0586 | Methylimidazoleacetic acid | 7.3 | 2.7E-03 | 1.7E-02 | Day2-Day13; Day2-Day18; Day7-Day2 |
| 7.3 | 149.0511 | L-Methionine | 8.2 | 1.6E-03 | 1.2E-02 | Day2-Day18; Day7-Day18 |
| 13.2 | 159.1259 | DL-2-Aminooctanoicacid | 7.0 | 3.3E-03 | 2.0E-02 | Day2-Day18; Day7-Day18 |
| **Tryptophan metabolism** | | | | | | |
| 7.3 | 117.0579 | Indole | 14.9 | 6.9E-05 | 1.0E-03 | Day2-Day13; Day7-Day13; Day2-Day18; Day7-Day18 |
| 5.4 | 145.0528 | 3-Methyleneoxindole | 10.8 | 4.0E-04 | 4.2E-03 | Day2-Day13; Day2-Day18; Day7-Day2 |
| 6.1 | 145.0528 | 3-Methyleneoxindole | 8.8 | 1.1E-03 | 8.7E-03 | Day2-Day13; Day2-Day18 |
| 10.7 | 145.0528 | 3-Methyleneoxindole | 5.1 | 1.2E-02 | 4.9E-02 | Day2-Day18; Day7-Day2 |
| 6.1 | 133.0528 | Indoxyl | 7.4 | 2.6E-03 | 1.7E-02 | Day2-Day13; Day7-Day2 |
| 7.0 | 149.0477 | 5,6-Dihydroxyindole | 11.6 | 2.8E-04 | 3.2E-03 | Day2-Day13; Day2-Day18; Day7-Day2 |
| 6.2 | 189.0426 | Kynurenate | 17.5 | 2.7E-05 | 5.5E-04 | Day2-Day13; Day2-Day18; Day7-Day18 |
| 10.1 | 177.0790 | 5-Hydroxytryptophol | 5.1 | 1.2E-02 | 4.9E-02 | Day2-Day18 |
| 9.5 | 191.0946 | 5-Methoxytryptophol | 21.6 | 7.1E-06 | 2.2E-04 | Day18-Day13; Day2-Day13; Day7-Day13; Day2-Day18; Day7-Day18 |
| 6.1 | 213.0096 | Indoxylsulfate | 7.6 | 2.2E-03 | 1.5E-02 | Day2-Day13; Day7-Day2 |

| | | | | | | |
|---|---|---|---|---|---|---|
| 9.3 | 219.1008 | tryptophanhydroxamate | 47.1 | 3.6E-08 | 2.2E-06 | Day2-Day13; Day7-Day13; Day2-Day18; Day7-Day18; Day7-Day2 |
| 7.1 | 229.0045 | 5-hydroxyindole sulfate or isomer | 9.1 | 9.3E-04 | 7.6E-03 | Day2-Day13; Day2-Day18; Day7-Day2 |
| 7.5 | 248.1161 | 6-Hydroxymelatonin | 18.0 | 2.2E-05 | 4.7E-04 | Day2-Day13; Day2-Day18; Day7-Day18; Day7-Day2 |
| **Guanidino compounds** | | | | | | |
| 9.6 | 113.0589 | Creatinine | 5.6 | 7.8E-03 | 3.8E-02 | Day2-Day13; Day2-Day18 |
| 15.5 | 117.0538 | Guanidinoacetate | 5.1 | 1.2E-02 | 4.9E-02 | Day2-Day18; Day7-Day2 |
| 14.5 | 131.0695 | Creatine | 6.5 | 4.5E-03 | 2.5E-02 | Day2-Day18; Day7-Day18 |
| 15.0 | 145.0851 | 4-Guanidinobutanoate | 5.2 | 1.1E-02 | 4.6E-02 | Day7-Day18 |
| 15.0 | 159.1008 | delta-Guanidinovaleric acid | 40.2 | 1.1E-07 | 5.9E-06 | Day2-Day13; Day7-Day13; Day2-Day18; Day7-Day18; Day7-Day2 |
| **Microbiome** | | | | | | |
| 4.6 | 94.0419 | Phenol | 8.0 | 1.8E-03 | 1.3E-02 | Day2-Day13; Day7-Day13; Day7-Day18 |
| 5.9 | 122.0368 | Benzoate | 5.5 | 8.4E-03 | 3.9E-02 | Day2-Day18 |
| 4.3 | 124.0524 | o-Methoxyphenol | 8.8 | 1.1E-03 | 8.7E-03 | Day2-Day13; Day2-Day18; Day7-Day2 |
| 4.6 | 173.9987 | Phenol sulfate | 6.8 | 3.6E-03 | 2.1E-02 | Day2-Day13; Day7-Day13 |
| 8.7 | 278.1267 | alpha-Ribazole | 17.4 | 2.7E-05 | 5.5E-04 | Day2-Day13; Day7-Day13; Day2-Day18; Day7-Day18 |
| **Peptides** | | | | | | |
| 11.6 | 212.1161 | Pro-Pro | 6.3 | 4.9E-03 | 2.6E-02 | Day2-Day13; Day7-Day13; Day2-Day18; Day7-Day18 |
| 10.1 | 228.1474 | Leu-Pro | 13.5 | 1.2E-04 | 1.5E-03 | Day2-Day13; Day2-Day18; Day7-Day2 |
| 12.9 | 228.1474 | Leu-Pro | 9.3 | 8.5E-04 | 7.1E-03 | Day2-Day13; Day2-Day18; Day7-Day2 |
| 10.4 | 231.1219 | Ala-Ala-Ala | 74.8 | 1.3E-09 | 4.9E-07 | Day2-Day13; Day2-Day18; Day7-Day2 |
| 13.1 | 231.1219 | Ala-Ala-Ala | 48.1 | 3.2E-08 | 2.1E-06 | Day2-Day13; Day7-Day13; Day2-Day18; Day7-Day18; Day7-Day2 |
| **Fatty acids lipids and metabolites** | | | | | | |
| 10.8 | 75.0684 | (R)-1-Aminopropan-2-ol | 9.9 | 6.2E-04 | 5.8E-03 | Day18-Day13; Day2-Day13 |
| 4.1 | 172.1463 | Decanoic acid | 9.4 | 8.3E-04 | 7.1E-03 | Day2-Day13; Day2-Day18; Day7-Day2 |
| 4.1 | 186.1620 | [FA (11:0)] undecanoic acid | 5.6 | 8.3E-03 | 3.9E-02 | Day2-Day13; Day2-Day18; Day7-Day2 |
| 4.0 | 200.1776 | Dodecanoic acid | 20.3 | 1.1E-05 | 2.8E-04 | Day2-Day13; Day2-Day18; Day7-Day2 |
| 8.2 | 219.1107 | pantothenic acid | 14.3 | 8.7E-05 | 1.2E-03 | Day2-Day13; Day2-Day18; Day7-Day2 |
| 3.8 | 270.2559 | heptadecanoic acid | 7.3 | 2.7E-03 | 1.7E-02 | Day2-Day13; Day2-Day18; Day7-Day2 |
| 3.9 | 276.2089 | octadecatetraenoic acid | 6.4 | 4.8E-03 | 2.6E-02 | Day2-Day18 |

| | | | | | | |
|---|---|---|---|---|---|---|
| 10.9 | 203.1158 | O-Acetylcarnitine | 8.0 | 1.8E-03 | 1.3E-02 | Day2-Day18; Day7-Day18 |
| 11.3 | 247.1420 | Hydroxybutyrylcarnitine | 5.6 | 8.2E-03 | 3.8E-02 | Day7-Day18 |
| 4.6 | 284.2715 | Octadecanoic acid | 5.9 | 6.5E-03 | 3.3E-02 | Day2-Day13; Day2-Day18; Day7-Day2 |
| 3.9 | 294.2195 | hydroxylinolenic acid | 5.5 | 8.9E-03 | 4.0E-02 | Day2-Day18 |
| 3.8 | 306.2559 | eicosatrienoic acid | 12.0 | 2.3E-04 | 2.7E-03 | Day2-Day13; Day2-Day18; Day7-Day2 |
| 3.8 | 308.2715 | eicosadienoic acid | 6.8 | 3.5E-03 | 2.0E-02 | Day2-Day13; Day2-Day18 |
| 4.0 | 336.2301 | dihydroxyeicosatetrenoic acid | 5.7 | 7.6E-03 | 3.8E-02 | Day2-Day13; Day7-Day13 |
| 4.0 | 338.2457 | dihydroxy eicostetrienoic acid | 11.5 | 2.9E-04 | 3.3E-03 | Day2-Day13; Day2-Day18 |
| 3.8 | 354.3134 | oxodocosanoic acid | 5.9 | 6.5E-03 | 3.3E-02 | Day2-Day13; Day2-Day18; Day7-Day2 |
| 3.7 | 356.2715 | [FA (24:6)] 4,8,12,15,19,21-tetracosahexaenoic acid | 10.6 | 4.4E-04 | 4.6E-03 | Day2-Day13; Day2-Day18; Day7-Day18 |
| 4.3 | 366.2042 | dihydroxyeicosaatetradiencoic acid | 6.8 | 3.6E-03 | 2.1E-02 | Day2-Day13; Day7-Day2 |
| 4.7 | 397.3192 | trans-Hexadec-2-enoylcarnitine | 6.1 | 5.7E-03 | 3.0E-02 | Day18-Day13; Day2-Day18; Day7-Day18 |
| 4.7 | 399.3349 | [FA] O-Palmitoyl-R-carnitine | 11.4 | 3.0E-04 | 3.3E-03 | Day18-Day13; Day2-Day18; Day7-Day18 |
| 4.8 | 420.2641 | LPA 18:0 | 5.1 | 1.1E-02 | 4.7E-02 | Day2-Day18 |
| 4.6 | 438.2746 | GP 18:0 | 89.5 | 3.3E-10 | 2.6E-07 | Day2-Day13; Day2-Day18; Day7-Day2 |
| 4.9 | 451.2699 | LPE 16:1 | 9.0 | 1.0E-03 | 8.2E-03 | Day2-Day18; Day7-Day2 |
| 4.8 | 453.2855 | LPE16:0 | 18.9 | 1.7E-05 | 4.0E-04 | Day2-Day13; Day2-Day18; Day7-Day2 |
| 3.6 | 466.3117 | Cholesterolsulfate | 5.0 | 1.2E-02 | 5.0E-02 | Day2-Day13; Day7-Day2 |
| 4.7 | 479.3012 | LPE18:1 | 6.1 | 5.8E-03 | 3.0E-02 | Day2-Day18; Day7-Day2 |
| 4.8 | 479.3376 | LPC 16:1 ether | 5.9 | 6.6E-03 | 3.3E-02 | Day2-Day13 |
| 4.9 | 480.2852 | GP 10:0 10:0 | 15.3 | 5.8E-05 | 9.6E-04 | Day2-Day13; Day2-Day18; Day7-Day2 |
| 4.9 | 481.3168 | LPE 18:0 | 23.1 | 4.6E-06 | 1.7E-04 | Day2-Day13; Day2-Day18; Day7-Day2 |
| 4.8 | 495.3325 | LPC16:0 | 7.7 | 2.2E-03 | 1.5E-02 | Day2-Day13; Day2-Day18; Day7-Day18 |
| 4.6 | 501.2855 | LPE20:4 | 6.3 | 5.1E-03 | 2.7E-02 | Day2-Day18; Day7-Day2 |
| 4.6 | 505.3532 | LPC18:2 ether | 5.5 | 8.5E-03 | 3.9E-02 | Day2-Day13; Day2-Day18 |
| 4.7 | 519.3325 | LPC18:2 | 15.5 | 5.5E-05 | 9.2E-04 | Day2-Day13; Day7-Day13; Day2-Day18; Day7-Day18 |
| 4.6 | 523.3638 | LPC18:0 | 62.6 | 4.6E-09 | 5.5E-07 | Day2-Day13; Day2-Day18; Day7-Day2 |
| 4.7 | 533.3482 | LPE 22:2 | 8.6 | 1.3E-03 | 9.8E-03 | Day2-Day13; Day2-Day18; Day7-Day18 |

| | | | | | | |
|---|---|---|---|---|---|---|
| 5.2 | 535.3274 | PC18:0 | 5.8 | 7.2E-03 | 3.6E-02 | Day2-Day13; Day2-Day18 |
| 4.6 | 535.3638 | LPE22:1 | 5.5 | 8.5E-03 | 3.9E-02 | Day2-Day13; Day2-Day18 |
| 4.6 | 547.3638 | LPC20:2 | 7.3 | 2.7E-03 | 1.7E-02 | Day7-Day18; Day7-Day2 |
| 4.6 | 549.3794 | LPC20:1 | 18.1 | 2.1E-05 | 4.7E-04 | Day2-Day13; Day2-Day18; Day7-Day2 |
| 5.0 | 565.3744 | PC 20:0 | 16.2 | 4.2E-05 | 7.5E-04 | Day2-Day13; Day2-Day18; Day7-Day2 |
| 4.6 | 569.3482 | LPC22:5 | 7.0 | 3.2E-03 | 1.9E-02 | Day2-Day18; Day7-Day2 |
| 4.3 | 572.2962 | LPI 16:0 | 9.1 | 9.3E-04 | 7.6E-03 | Day2-Day13; Day2-Day18; Day7-Day2 |
| 4.5 | 577.4108 | LPC22:1 | 7.2 | 2.9E-03 | 1.8E-02 | Day2-Day13; Day2-Day18 |
| 4.1 | 672.4730 | GP34:2 | 8.6 | 1.3E-03 | 9.8E-03 | Day2-Day13; Day2-Day18 |
| 4.1 | 696.4730 | GP36:4 | 8.9 | 1.1E-03 | 8.4E-03 | Day2-Day13; Day2-Day18; Day7-Day2 |
| 4.2 | 705.5309 | PC30:0 | 9.8 | 6.5E-04 | 6.0E-03 | Day2-Day13; Day2-Day18; Day7-Day2 |
| 4.4 | 730.5989 | SM(d18:0/18:1) | 14.9 | 6.7E-05 | 1.0E-03 | Day2-Day13; Day2-Day18; Day7-Day18; Day7-Day2 |
| 4.1 | 731.5465 | PC32:1 | 9.4 | 8.2E-04 | 7.1E-03 | Day2-Day13; Day2-Day18 |
| 4.1 | 759.5778 | PC34:1 | 5.6 | 8.0E-03 | 3.8E-02 | Day2-Day13; Day2-Day18 |
| 4.1 | 781.5627 | PC36:4 | 5.7 | 7.6E-03 | 3.8E-02 | Day2-Day18 |
| 4.1 | 785.5935 | PC36:2 | 5.6 | 7.9E-03 | 3.8E-02 | Day18-Day13; Day2-Day18 |
| 4.1 | 787.6091 | PC 36:1 | 13.9 | 1.0E-04 | 1.4E-03 | Day2-Day13; Day2-Day18; Day7-Day2 |
| 4.0 | 793.5985 | PC 38:5 | 5.2 | 1.1E-02 | 4.6E-02 | Day2-Day13; Day7-Day2 |
| 4.0 | 809.5935 | PC38:4 | 12.8 | 1.6E-04 | 2.0E-03 | Day2-Day13; Day2-Day18; Day7-Day2 |
| 4.2 | 811.5363 | PS 38:4 | 13.9 | 1.0E-04 | 1.4E-03 | Day2-Day13; Day2-Day18; Day7-Day18; Day7-Day2 |
| 4.2 | 813.5520 | PS 38:3 | 62.3 | 4.8E-09 | 5.5E-07 | Day2-Day13; Day7-Day13; Day2-Day18; Day7-Day2 |
| 4.0 | 813.6248 | PC 38:2 | 25.7 | 2.4E-06 | 8.8E-05 | Day18-Day13; Day2-Day13; Day2-Day18; Day7-Day18; Day7-Day2 |
| 4.2 | 817.5833 | PS 38:1 | 5.3 | 1.0E-02 | 4.5E-02 | Day2-Day18 |
| 4.0 | 833.5935 | PC40:6 | 21.9 | 6.5E-06 | 2.2E-04 | Day2-Day13; Day7-Day13; Day2-Day18; Day7-Day18; Day7-Day2 |
| 4.0 | 835.6091 | PC 40:5 | 7.7 | 2.1E-03 | 1.5E-02 | Day2-Day13; Day2-Day18 |
| 3.8 | 836.5415 | PI 34:1 | 10.8 | 4.0E-04 | 4.2E-03 | Day2-Day13; Day2-Day18; Day7-Day2 |
| 4.2 | 841.5833 | PS 40:3 | 5.2 | 1.0E-02 | 4.6E-02 | Day2-Day18; Day7-Day2 |
| 3.7 | 858.5258 | PI 26:4 | 13.7 | 1.1E-04 | 1.4E-03 | Day2-Day13; Day2-Day18; Day7-Day2 |
| 4.2 | 867.5989 | PS 42:4 | 7.2 | 2.8E-03 | 1.7E-02 | Day18-Day13; Day2-Day18; Day7-Day18 |
| **Miscellaneous** | | | | | | |

| | | | | | | |
|---|---|---|---|---|---|---|
| 12.3 | 83.0735 | Piperideine | 5.5 | 8.7E-03 | 4.0E-02 | Day7-Day18 |
| 3.7 | 97.9674 | Sulfate | 13.6 | 1.1E-04 | 1.5E-03 | Day2-Day13; Day2-Day18; Day7-Day2 |
| 4.3 | 97.9674 | Sulfate | 11.9 | 2.4E-04 | 2.7E-03 | Day2-Day13; Day2-Day18; Day7-Day2 |
| 13.2 | 100.0524 | Tiglic acid | 14.9 | 6.8E-05 | 1.0E-03 | Day2-Day13; Day7-Day13; Day2-Day18; Day7-Day18 |
| 6.7 | 80.9646 | HSO3- | 5.2 | 1.1E-02 | 4.6E-02 | Day2-Day13 |
| 13.1 | 102.0317 | 2-Oxobutanoate | 5.2 | 1.1E-02 | 4.6E-02 | Day7-Day18 |
| 5.9 | 110.0480 | Imidazole-4-acetaldehyde | 6.8 | 3.6E-03 | 2.1E-02 | Day2-Day13; Day2-Day18 |
| 5.5 | 113.0841 | epsilon-Caprolactam | 9.5 | 7.7E-04 | 6.9E-03 | Day2-Day13; Day2-Day18; Day7-Day2 |
| 21.9 | 130.1106 | N-Acetylputrescine | 6.9 | 3.3E-03 | 2.0E-02 | Day2-Day13; Day2-Day18; Day7-Day18 |
| 25.7 | 131.1059 | N-Carbamoylputrescine | 12.7 | 1.7E-04 | 2.1E-03 | Day2-Day13; Day7-Day13; Day2-Day18; Day7-Day18 |
| 8.2 | 139.0382 | 2-amino-4-carboxypyrimidine | 9.3 | 8.7E-04 | 7.2E-03 | Day2-Day13; Day2-Day18; Day7-Day2 |
| 13.2 | 145.1103 | 4-Trimethylammoniobutanoate | 16.2 | 4.2E-05 | 7.5E-04 | Day2-Day13; Day2-Day18; Day7-Day18; Day7-Day2 |
| 10.9 | 84.0211 | 4-Hydroxy-2-butynal | 7.8 | 1.9E-03 | 1.4E-02 | Day2-Day18; Day7-Day18 |
| 11.3 | 123.0433 | Pyrazinamide | 20.3 | 1.1E-05 | 2.8E-04 | Day2-Day13; Day2-Day18; Day7-Day2 |
| 11.3 | 124.0637 | Methylimidazole acetaldehyde | 43.6 | 6.3E-08 | 3.5E-06 | Day2-Day13; Day2-Day18; Day7-Day2 |
| 11.9 | 79.9262 | Br- | 5.8 | 7.1E-03 | 3.6E-02 | Day2-Day13; Day7-Day13 |
| 3.7 | 584.2635 | Bilirubin | 6.4 | 4.6E-03 | 2.5E-02 | Day2-Day18; Day7-Day18 |

**Mothers**

ANOVA with Tukey's test and FDR values indicating metabolites which change significantly between the days

| RT min | MW | Metabolite | f.value | p.value | FDR | Tukey's HSD |
|---|---|---|---|---|---|---|
| **Amino acids and their metabolites** | | | | | | |
| 15.4 | 75.0320 | Glycine | 9.0 | 1.00E-03 | 2.03E-02 | Day2-Day13; Day2-Day18; Day7-Day2 |
| 14.5 | 89.0477 | L-Alanine | 8.7 | 1.20E-03 | 2.37E-02 | Day2-Day13; Day2-Day18; Day7-Day2 |
| 8.9 | 131.0582 | L-Glutamate 5-semialdehyde | 16.2 | 4.18E-05 | 2.37E-03 | Day2-Day13; Day2-Day18; Day7-Day2 |
| 15.0 | 145.0851 | 4-Guanidinobutanoate | 7.8 | 1.99E-03 | 3.22E-02 | Day2-Day13; Day7-Day13; Day2-Day18; Day7-Day18 |
| 14.0 | 147.0532 | L-Glutamate | 10.6 | 4.51E-04 | 1.23E-02 | Day2-Day13; Day2-Day18 |
| 7.1 | 159.0684 | Indole-3-acetaldehyde | 11.8 | 2.54E-04 | 8.41E-03 | Day2-Day13; Day7-Day13; Day2-Day18; Day7-Day18 |
| 8.0 | 159.0895 | 5-Acetamidopentanoate | 13.2 | 1.33E-04 | 6.21E-03 | Day2-Day13; Day2-Day18; Day7-Day18 |
| 10.5 | 160.0848 | D-Alanyl-D-alanine | 8.1 | 1.62E-03 | 2.80E-02 | Day2-Day13; Day2-Day18; Day7-Day18 |
| 14.1 | 173.0800 | 5-Guanidino-2-oxopentanoate | 11.8 | 2.49E-04 | 8.41E-03 | Day2-Day13; Day7-Day13; Day2-Day18; Day7-Day18 |
| 10.0 | 189.1001 | (Carboxyethyl]amino}pentanoate | 7.5 | 2.38E-03 | 3.75E-02 | Day7-Day13; Day7-Day18 |
| **Pyrimidines** | | | | | | |
| 9.1 | 111.0433 | Cytosine | 7.4 | 2.57E-03 | 3.81E-02 | Day2-Day13; Day2-Day18; Day7-Day2 |
| 8.3 | 126.0429 | Thymine | 6.8 | 3.71E-03 | 4.84E-02 | Day2-Day13; Day2-Day18 |
| **Carnitines** | | | | | | |
| 6.6 | 285.1940 | 2-Octenoylcarnitine | 11.0 | 3.55E-04 | 1.08E-02 | Day2-Day13; Day2-Day18; Day7-Day2 |
| 4.7 | 397.3192 | Hexadecenoylcarnitine | 8.6 | 1.23E-03 | 2.37E-02 | Day2-Day13; Day2-Day18 |
| 4.6 | 425.3505 | Elaidiccarnitine | 9.1 | 9.30E-04 | 1.94E-02 | Day2-Day13; Day2-Day18; Day7-Day18 |
| **Lipids and metabolites** | | | | | | |
| 3.9 | 248.1776 | hexadecatetraenoic acid | 6.8 | 3.56E-03 | 4.78E-02 | Day2-Day13; Day7-Day13; Day2-Day18; Day7-Day18 |
| 3.9 | 312.2665 | oxo-nonadecanoic acid | 10.8 | 4.08E-04 | 1.17E-02 | Day2-Day13; Day7-Day13; Day2-Day18; Day7-Day18 |
| 4.5 | 348.1937 | Trihydroxyeicosatrienoic acid | 7.9 | 1.85E-03 | 3.09E-02 | Day2-Day13; Day7-Day13; Day2-Day18; Day7-Day18 |
| 4.3 | 350.2093 | Trihydroxyeicosadienoic acid | 18.7 | 1.77E-05 | 1.56E-03 | Day2-Day13; Day7-Day13; Day2-Day18; Day7-Day18 |
| 4.8 | 422.2797 | LPA 18:0 | 32.0 | 5.43E-07 | 2.15E-04 | Day2-Day13; Day7-Day13; Day2-Day18; Day7-Day18 |

| | | | | | | |
|---|---|---|---|---|---|---|
| 4.8 | 479.3012 | LPE 18:0 | 9.5 | 7.57E-04 | 1.62E-02 | Day2-Day18; Day7-Day18 |
| 4.9 | 481.3532 | LPC 16:2 | 12.6 | 1.78E-04 | 7.08E-03 | Day2-Day13; Day7-Day13; Day2-Day18; Day7-Day18 |
| 4.7 | 509.3481 | LPE 18:0 | 28.8 | 1.11E-06 | 2.93E-04 | Day2-Day13; Day7-Day13; Day2-Day18; Day7-Day18 |
| 4.3 | 572.2962 | LPI 16:0 | 7.9 | 1.87E-03 | 3.09E-02 | Day2-Day13; Day2-Day18; Day7-Day2 |
| 4.4 | 617.4784 | SP 16:0 | 21.4 | 7.53E-06 | 9.95E-04 | Day2-Day13; Day2-Day18; Day7-Day18; Day7-Day2 |
| 4.4 | 674.5363 | SM(d18:1/14:0) | 11.8 | 2.50E-04 | 8.41E-03 | Day2-Day13; Day2-Day18; Day7-Day2 |
| 4.4 | 688.5519 | SM (d18:1/15:0) | 8.6 | 1.25E-03 | 2.37E-02 | Day2-Day18 |
| 4.1 | 696.4730 | GP 36:4 | 6.8 | 3.72E-03 | 4.84E-02 | Day18-Day13; Day2-Day18; Day7-Day18 |
| 4.4 | 702.5670 | SM (d18:1/15:0) | 20.5 | 1.00E-05 | 1.13E-03 | Day18-Day13; Day2-Day13; Day2-Day18; Day7-Day18; Day7-Day2 |
| 4.4 | 730.5989 | SM(d18:1/18:0) | 21.6 | 7.07E-06 | 9.95E-04 | Day2-Day13; Day7-Day13; Day2-Day18; Day7-Day18; Day7-Day2 |
| 4.1 | 781.5627 | PC 36:4 | 8.4 | 1.43E-03 | 2.52E-02 | Day18-Day13; Day2-Day18; Day7-Day18 |
| 4.0 | 791.5829 | PC 38:6 ether | 12.3 | 1.98E-04 | 7.49E-03 | Day2-Day13; Day7-Day13; Day2-Day18; Day7-Day18 |
| 4.0 | 793.5985 | PC 38:5 ether | 8.4 | 1.38E-03 | 2.49E-02 | Day2-Day13; Day2-Day18 |
| 4.0 | 807.5778 | PC 38:5 | 7.3 | 2.60E-03 | 3.81E-02 | Day2-Day18; Day7-Day18 |
| 4.4 | 812.6771 | SM d18:1/24:1(15Z) | 10.5 | 4.74E-04 | 1.25E-02 | Day2-Day13; Day2-Day18; Day7-Day2 |
| 3.7 | 858.5258 | PI 36:4 | 9.9 | 6.38E-04 | 1.45E-02 | Day2-Day13; Day2-Day18; Day7-Day2 |
| **Miscellaneous** | | | | | | |
| 4.0 | 96.0575 | Cyclohex-2-enone | 7.0 | 3.13E-03 | 4.36E-02 | Day7-Day13; Day7-Day18 |
| 4.6 | 94.0419 | Phenol | 9.8 | 6.74E-04 | 1.48E-02 | Day2-Day13; Day2-Day18; Day7-Day2 |
| 5.1 | 110.0368 | p-Benzenediol | 7.4 | 2.55E-03 | 3.81E-02 | Day7-Day13; Day7-Day18 |
| 5.5 | 113.0841 | epsilon-Caprolactam | 10.3 | 5.01E-04 | 1.28E-02 | Day2-Day13; Day2-Day18; Day7-Day2 |
| 4.1 | 272.2140 | 3-Oxo-delta4-steroid | 6.7 | 3.95E-03 | 4.98E-02 | Day2-Day18; Day7-Day18 |

**Figure S3A. OPLS plots of pups predicted versus actual day based on six metabolites. CVANOVA 1.4 x $10^{-7}$.**

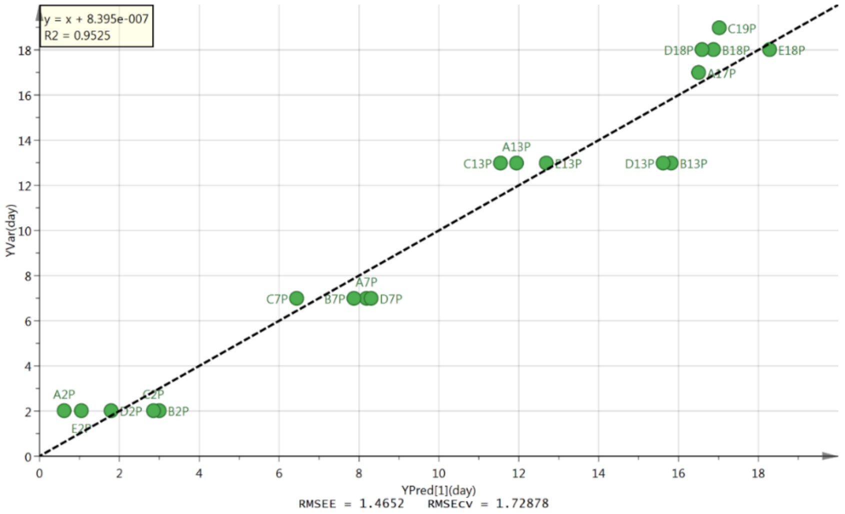


**Figure S3B. Loadings plot for OPLS plot for pups of observed versus predicted day based on six metabolites.**

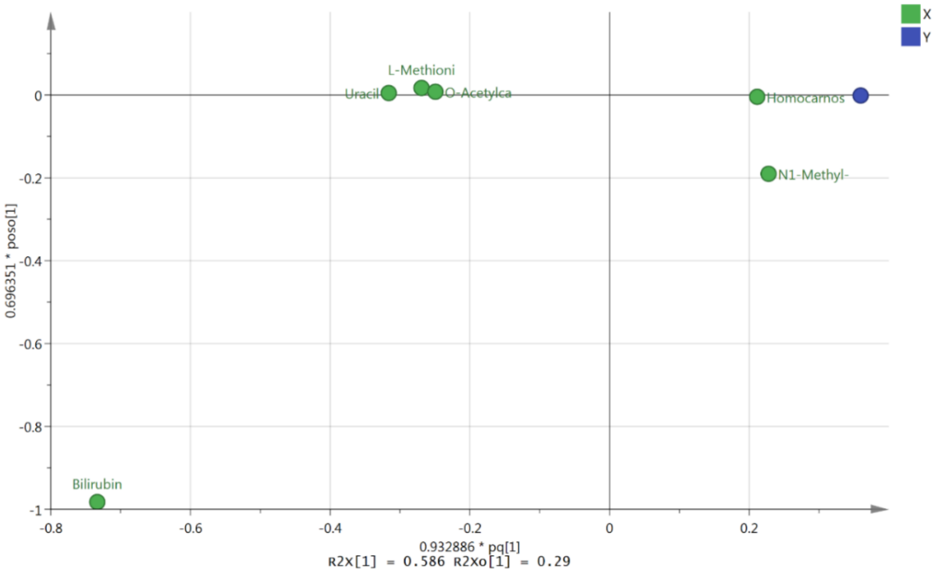

**Figure S4. Reciprocal trends in serum levels of bilirubin in pup and mother grey seals with time after birth.** Pups – suffix 'P'. Mothers – suffix 'M'.

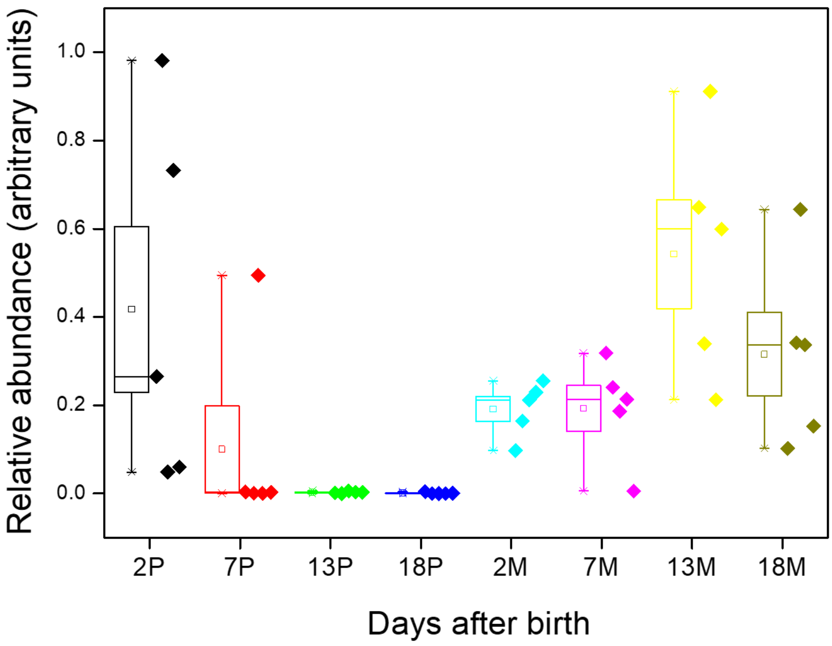

**Figure S5. Examples of compounds exhibiting relative stability with time, and diversity in levels, between individual pup.**

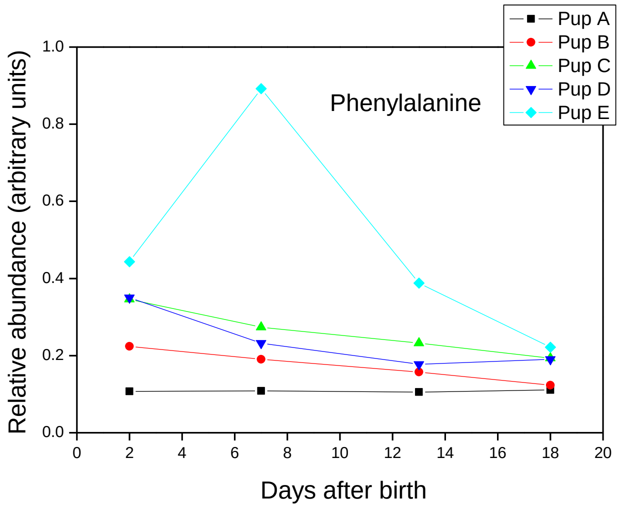


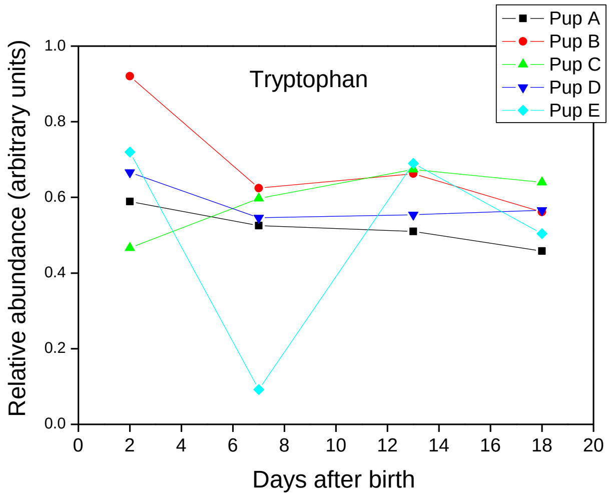


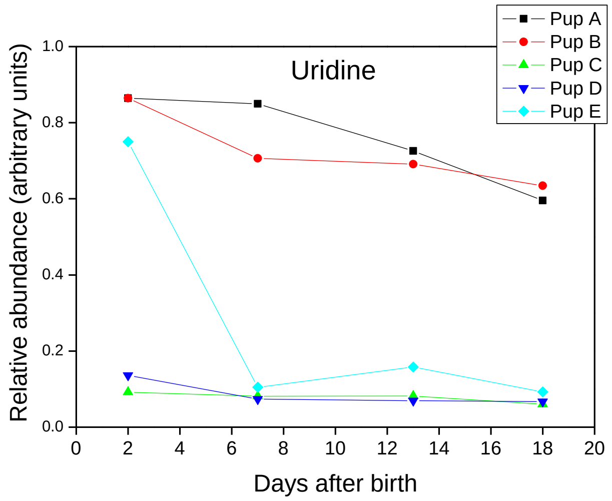

**Figure S6. Changes in metabolites related to energy metabolism in grey seal milk with time after birth.** Comparable trends occur in the sera of pups - see Figure 3 of main paper. Box and whisker settings - line in box, median; square in box, mean; box boundaries, standard error; crosses and whisker bars, range. Taken from ref. [2].

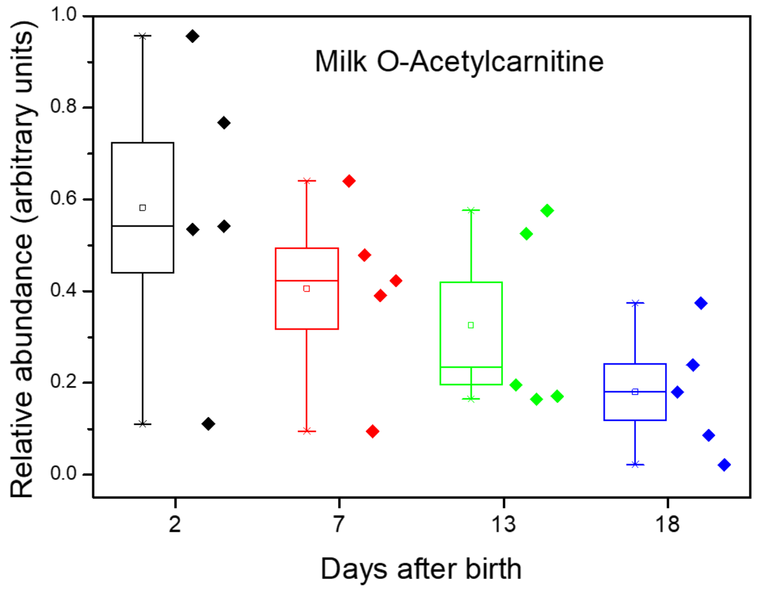


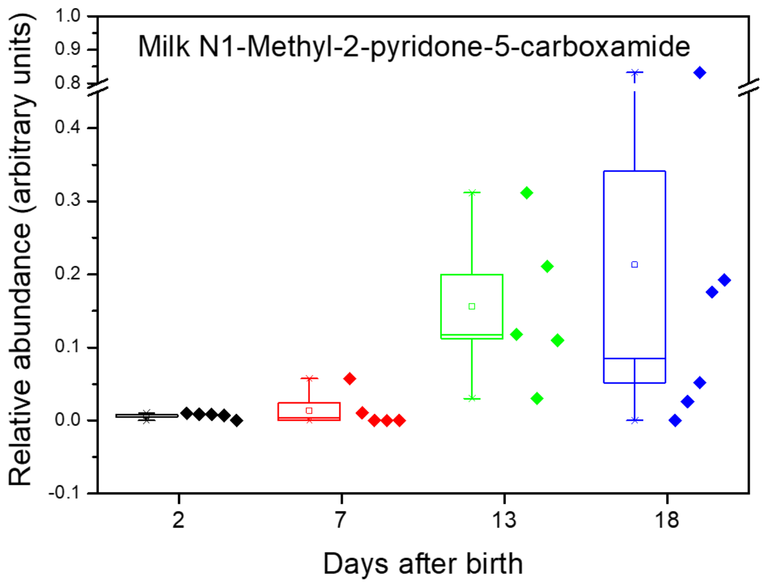

**Figure S7. The top 51 metabolites in serum by abundance from soon after birth to imminent weaning in Atlantic grey seal mothers.** Column codes - the initial letter is the seal mother/pup pair (A to E), the numeral is the days after birth, then 'M' for mother. Relative abundance/signal magnitude colour codes, red = 1 X $10^9$, yellow = 5 X $10^7$, to blue = 2 X $10^7$. M/Z, mass to charge ratio. m/z, mass divided by charge. Rt, retention times on the ZICpHILIC columns.

## Mothers

| M/Z | Rt (min) | **Chemical Formula** | **Metabolite name** | A2 M | B2 M | C2 M | D2 M | E2 M | A7 M | B7 M | C7 M | D7 M | E7 M | A13 M | B13 M | C13 M | D13 M | E13 M | A17 M | B18 M | C19 M | D18 M | E18 M |
|---|---|---|---|---|---|---|---|---|---|---|---|---|---|---|---|---|---|---|---|---|---|---|---|
| 496.340 | 4.8 | C24H50NO7P | LPC 16:0 | | | | | | | | | | | | | | | | | | | | |
| 146.092 | 14.9 | C5H11N3O2 | 4-Guanidinobutanoate | | | | | | | | | | | | | | | | | | | | |
| 524.371 | 4.6 | C26H54NO7P | LPC18:0 | | | | | | | | | | | | | | | | | | | | |
| 132.077 | 14.4 | C4H9N3O2 | Creatine | | | | | | | | | | | | | | | | | | | | |
| 114.066 | 9.6 | C4H7N3O | Creatinine | | | | | | | | | | | | | | | | | | | | |
| 162.112 | 13.1 | C7H15NO3 | L-Carnitine | | | | | | | | | | | | | | | | | | | | |
| 522.355 | 4.7 | C26H52NO7P | LPC18:1 | | | | | | | | | | | | | | | | | | | | |
| 116.071 | 12.6 | C5H9NO2 | L-Proline | | | | | | | | | | | | | | | | | | | | |
| 309.280 | 3.8 | C20H38O2 | Eicosanoic acid | | | | | | | | | | | | | | | | | | | | |
| 127.050 | 11.3 | C5H6N2O2 | Thymine | | | | | | | | | | | | | | | | | | | | |
| 204.123 | 10.9 | C9H18NO4 | O-Acetylcarnitine | | | | | | | | | | | | | | | | | | | | |
| 782.570 | 4.1 | C44H81NO8P | PC36:4 | | | | | | | | | | | | | | | | | | | | |
| 409.236 | 4.8 | C19H39O7P | LPG16:0 | | | | | | | | | | | | | | | | | | | | |
| 301.218 | 3.8 | C20H30O2 | Eicosapentenoic acid | | | | | | | | | | | | | | | | | | | | |
| 550.387 | 4.6 | C28H56NO7P | LPC20:1 | | | | | | | | | | | | | | | | | | | | |
| 780.554 | 4.1 | C44H78NO8P | PC36:7 | | | | | | | | | | | | | | | | | | | | |
| 337.312 | 3.7 | C22H42O2 | Docasenoic acid | | | | | | | | | | | | | | | | | | | | |
| 808.585 | 4.0 | C46H82NO8P | PC38:5 | | | | | | | | | | | | | | | | | | | | |
| 141.066 | 9.1 | C6H8N2O2 | Methylimidazoleacetic acid | | | | | | | | | | | | | | | | | | | | |
| 118.086 | 12.4 | C5H11NO2 | L-Valine | | | | | | | | | | | | | | | | | | | | |
| 810.601 | 4.0 | C46H84NO8P | PC38:4 | | | | | | | | | | | | | | | | | | | | |

| | | | |
|---|---|---|---|
| 435.252 | 4.8 | C21H41O7P | LPA18:1 |
| 760.586 | 4.1 | C42H82NO8P | PC34:1 |
| 258.110 | 14.2 | C8H20NO6P | GPC |
| 494.324 | 4.9 | C24H48NO7P | LPC16:0 |
| 544.340 | 4.7 | C28H50NO7P | LPC20:4 |
| 170.092 | 12.7 | C7H11N3O2 | N(pi)-Methyl-L-histidine |
| 128.071 | 10.3 | C6H9NO2 | Tetrahydropyridine-2-carboxylate |
| 329.249 | 3.8 | C22H34O2 | Docosatetraenoic acid |
| 468.308 | 5.0 | C22H46NO7P | LPC14:0 |
| 437.268 | 4.6 | C21H43O7P | LPC18:0 |
| 303.233 | 3.8 | C20H32O2 | Eicosatetraenoic acid |
| 758.570 | 4.1 | C42H80NO8P | PC34:2 |
| 279.233 | 3.8 | C18H32O2 | Linoleate |
| 510.356 | 4.7 | C25H52NO7P | LPE20:0 |
| 542.324 | 4.7 | C28H48NO7P | LPC20:5 |
| 90.055 | 14.5 | C3H7NO2 | L-Alanine |
| 806.570 | 4.1 | C46H80NO8P | PC38:6 |
| 174.087 | 14.1 | C6H11N3O3 | 5-Guanidino-2-oxopentanoate |
| 275.202 | 3.8 | C18H28O2 | Octadectetraenoic acid |
| 520.340 | 4.8 | C26H50NO7P | LOPC18:2 |
| 786.601 | 4.1 | C44H84NO8P | PC36:2 |
| 132.102 | 10.7 | C6H13NO2 | L-Leucine |
| 784.585 | 4.1 | C44H82NO8P | PC36:3 |
| 258.108 | 11.3 | C10H15N3O5 | 5-Methylcytidine |
| 482.324 | 4.9 | C23H48NO7P | LPE18:0 |
| 277.217 | 3.8 | C18H30O2 | Octadecatrienoic acid |
| 703.575 | 4.4 | C39H79N2O6P | SP16:0 |
| 132.102 | 11.2 | C6H13NO2 | Isoleucine |

| | | | | | | | | | | | | | | | | | | | | | | | |
|---|---|---|---|---|---|---|---|---|---|---|---|---|---|---|---|---|---|---|---|---|---|---|---|
| 166.086 | 10.0 | C9H11NO2 | L-Phenylalanine | | | | | | | | | | | | | | | | | | | | |
| 124.008 | 14.4 | C2H7NO3S | Taurine | | | | | | | | | | | | | | | | | | | | |

**Figure S8A. OPLS plots showing strong fit between mothers' samples and day after birth based on six metabolites (**leucine, adenosine, nicotinamide, sulfocatachol, N-(hexadecanoyl)-sphing-4-enine-1-phosphocholine (SP16:0), and eicosanoic acid)**.**

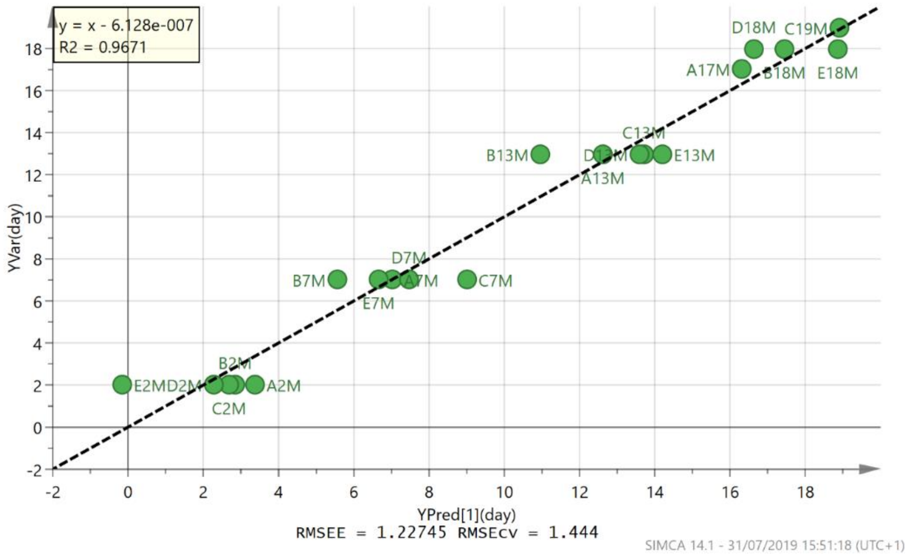


**Figure S8B. Loadings plot for OPLS plot for mothers of observed versus predicted day.**

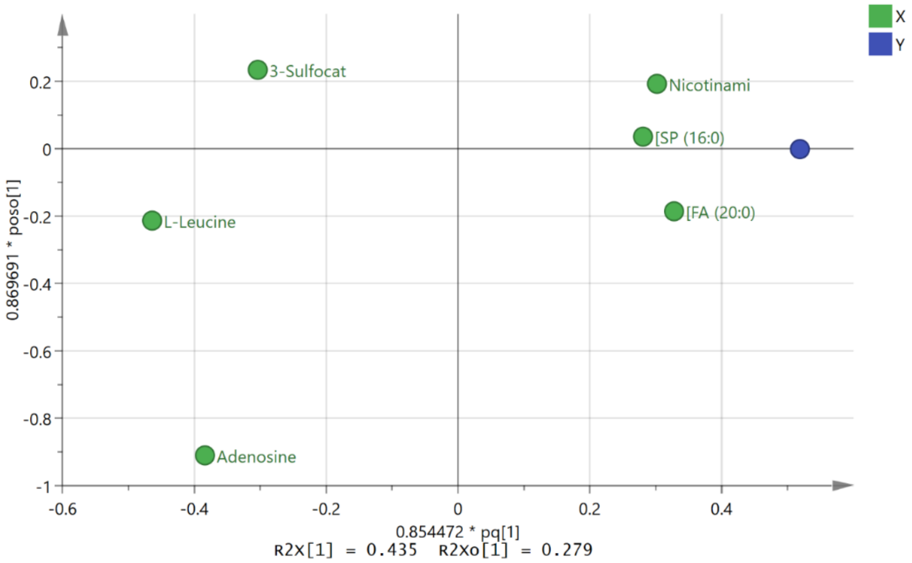

**Figure S9. Detailed box and whisker plots of the data presented as bar charts in Figure 5a (essential amino acids) and Figure 5b (conditionally amino acids) of the main text.** Box and whisker settings - line in box, median; square in box, mean; box boundaries, standard error; whisker bars, range. The scatter points refer to values for individual pup:mother ratio values. The days after birth labelled 18 are a combination of samples taken at 17, 18, or 19 days. The data on N6-Methyl-L-lysine that were not included in Figure 5a of the main text is presented here.

**(a) Essential amino acids**

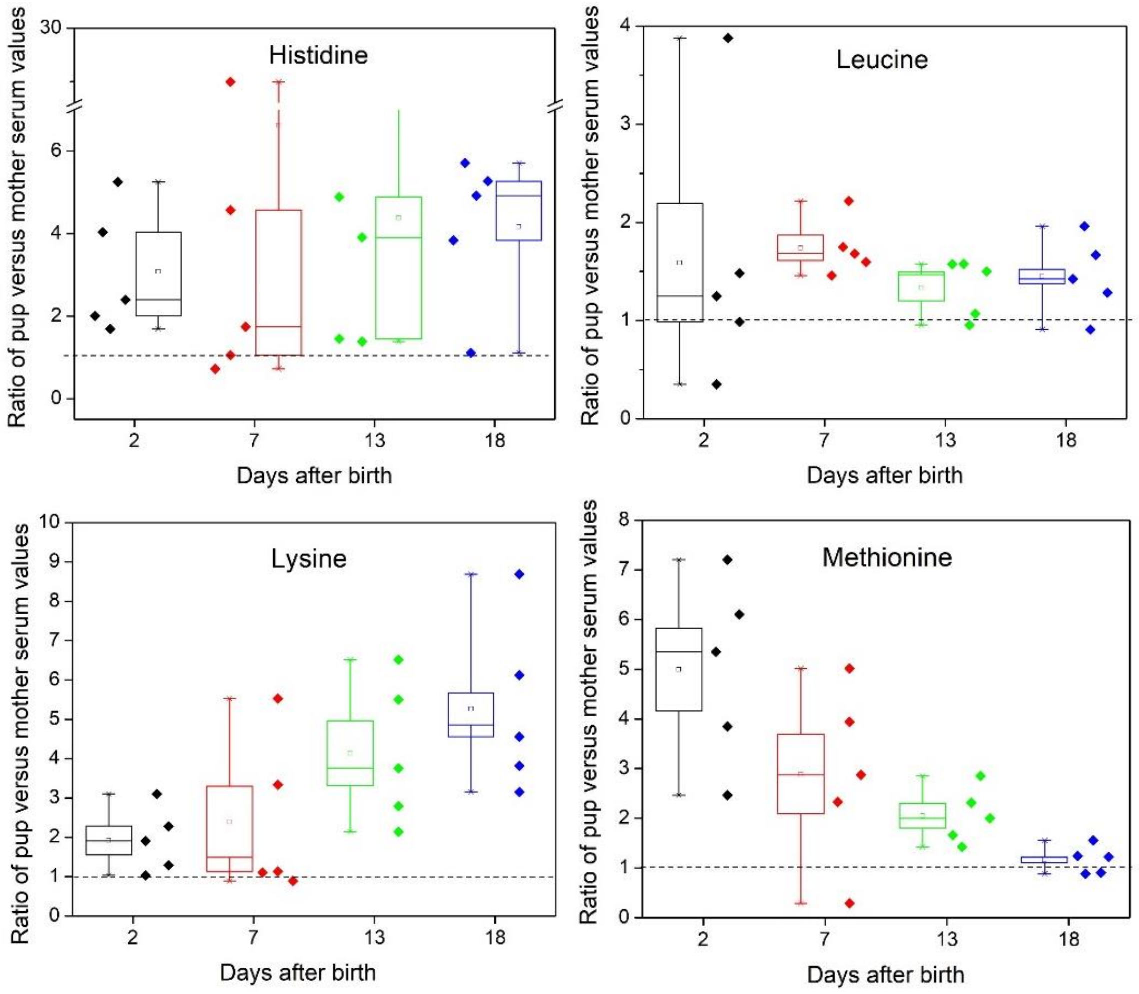

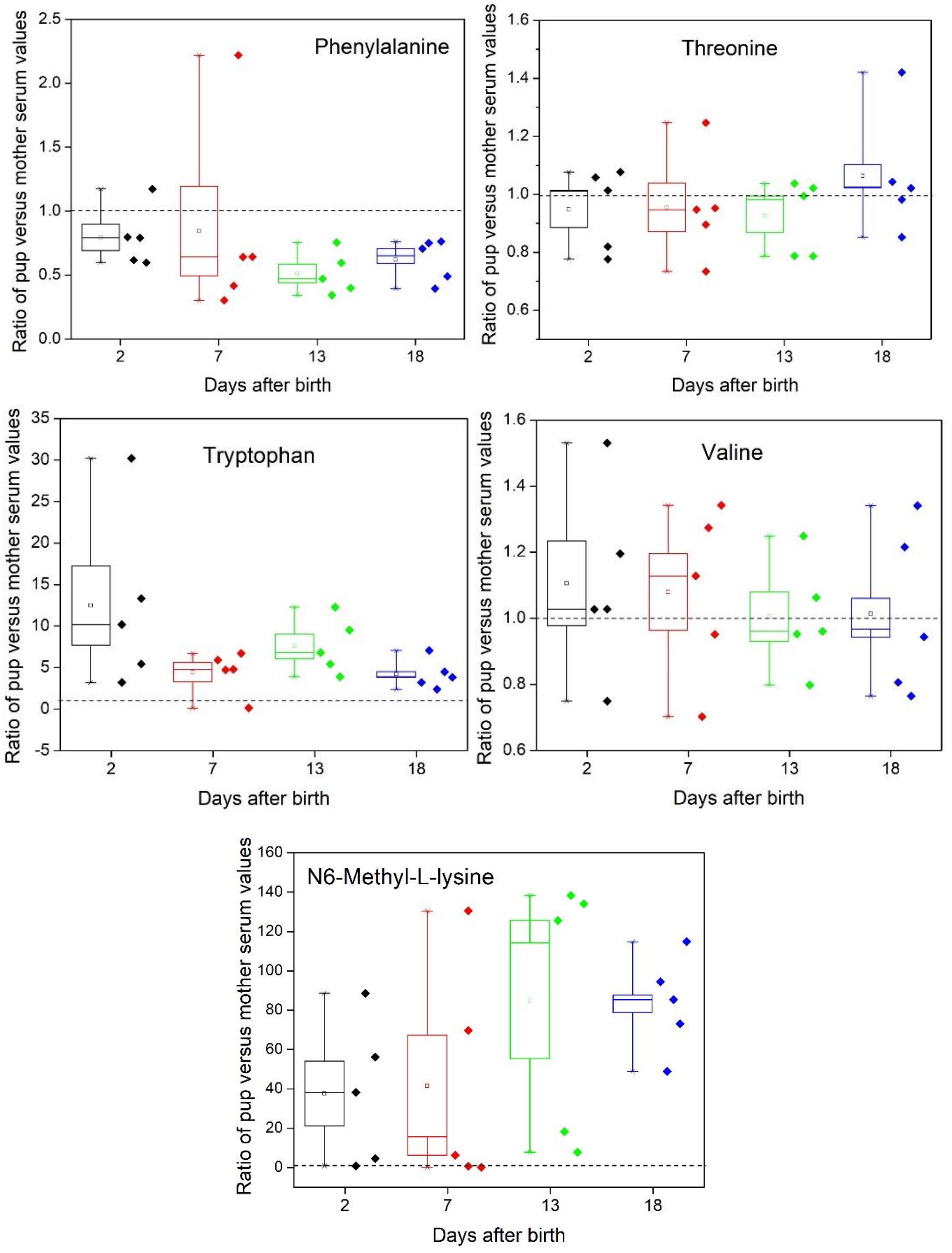
Phenylalanine
Ratio of pup versus mother serum values
0.0
0.5
1.0
1.5
2.0
2.5
2
7
13
18
Days after birth
Threonine
Ratio of pup versus mother serum values
0.6
0.8
1.0
1.2
1.4
1.6
2
7
13
18
Days after birth
Tryptophan
Ratio of pup versus mother serum values
-5
0
5
10
15
20
25
30
35
2
7
13
18
Days after birth
Valine
Ratio of pup versus mother serum values
0.6
0.8
1.0
1.2
1.4
1.6
2
7
13
18
Days after birth
N6-Methyl-L-lysine
Ratio of pup versus mother serum values
0
20
40
60
80
100
120
140
160
2
7
13
18
Days after birth

**(b) Conditionally essential amino acids**

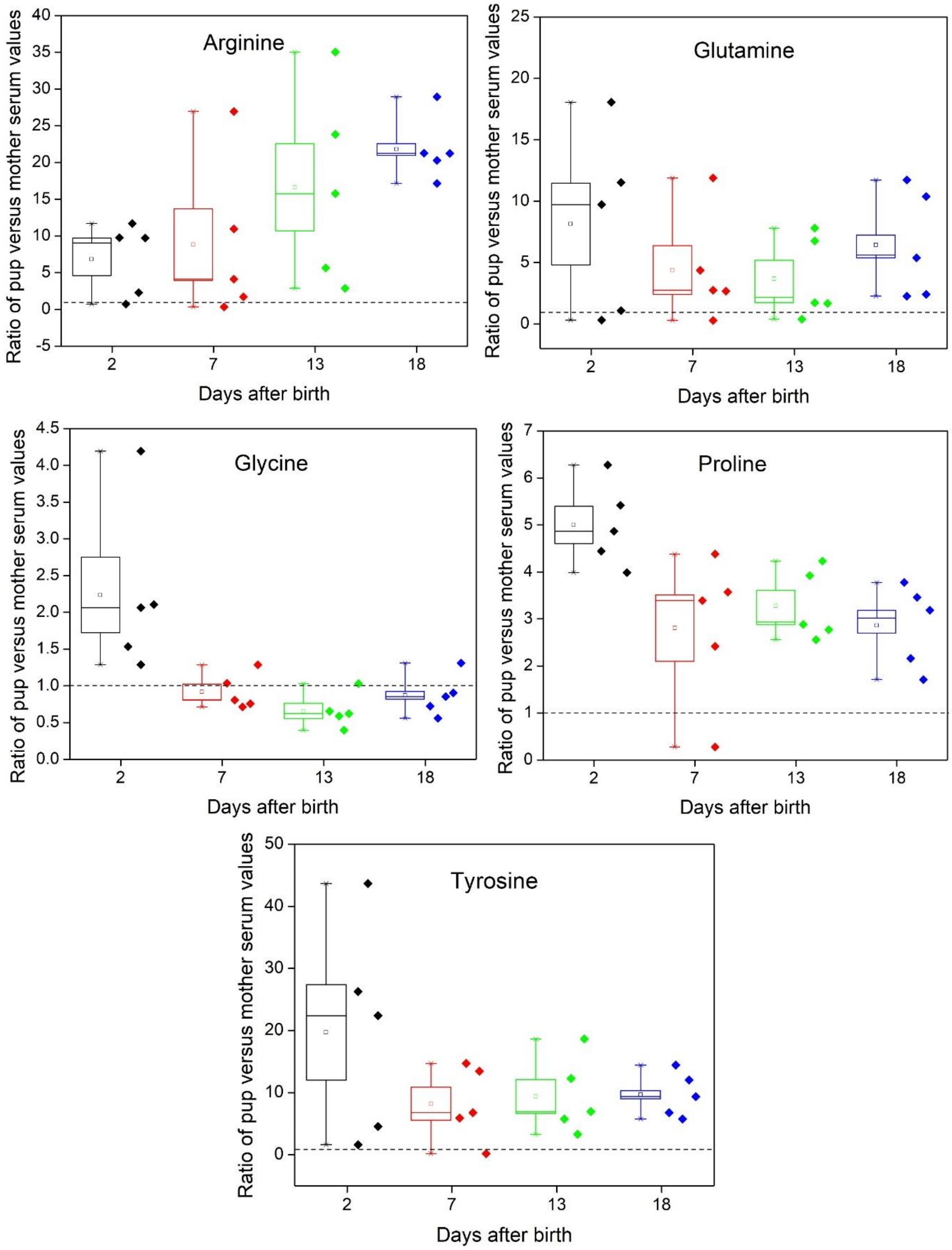

**Figure S10. Detailed box and whisker plots of the data presented on selected compounds as bar charts in Figure 6 of the main text.** Box and whisker settings - line in box, median; square in box, mean; box boundaries, standard error; whisker bars, range. The scatter points refer to values for individual pups:mother ratios. The days after birth labelled 18 are a combination of samples taken at 17, 18, or 19 days.

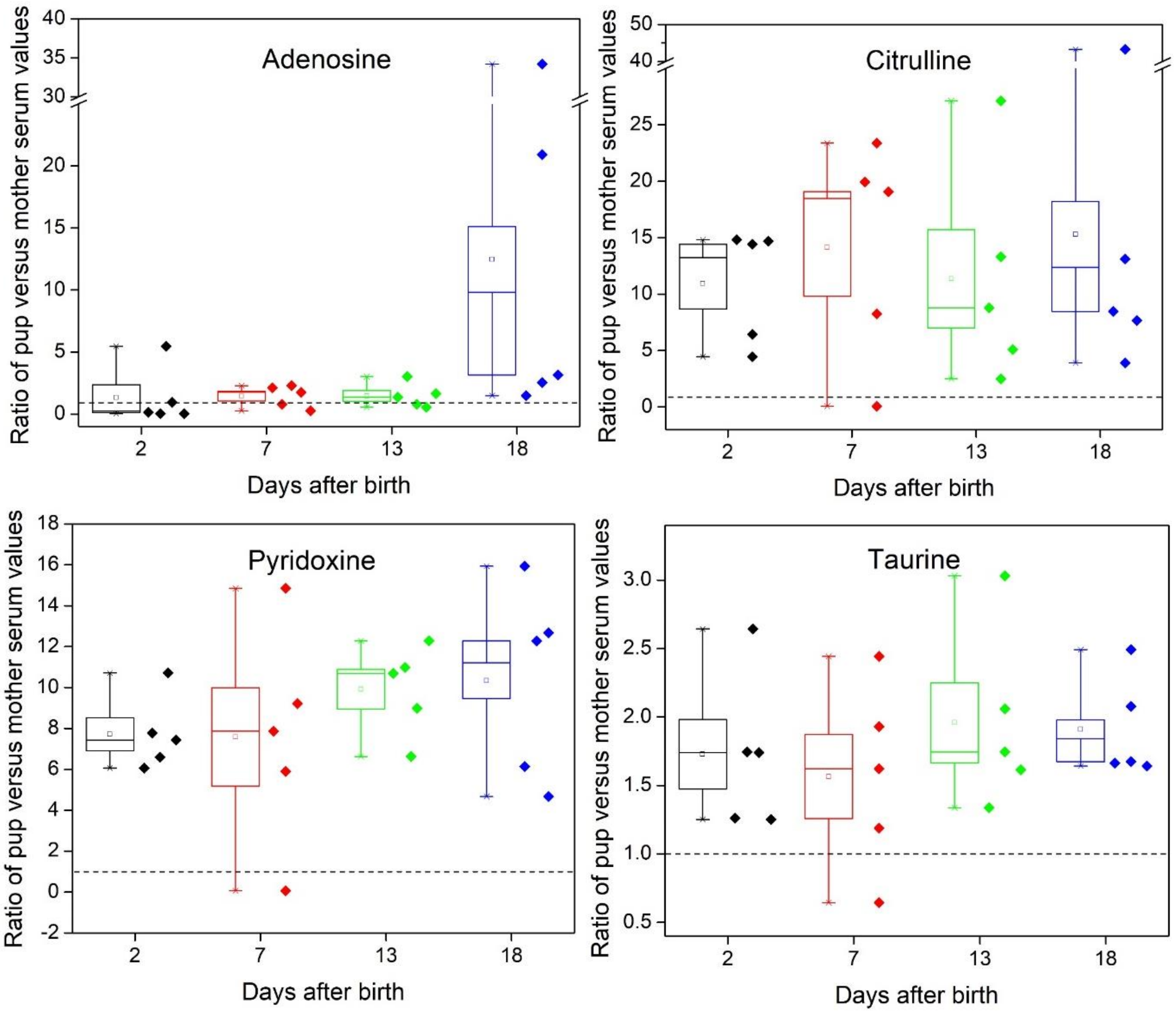

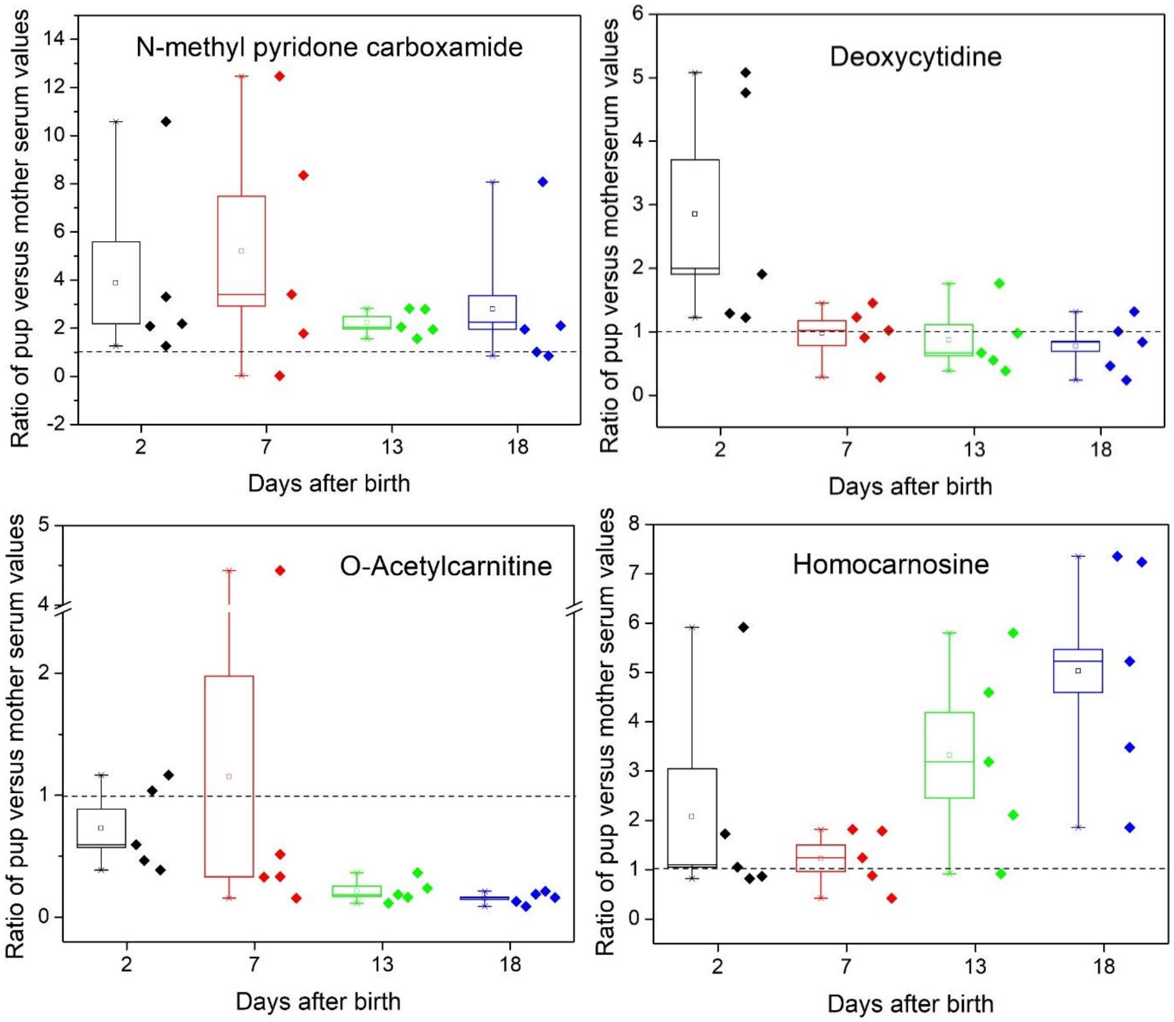
N-methyl pyridone carboxamide
Ratio of pup versus mother serum values
Days after birth
Deoxycytidine
Ratio of pup versus motherserum values
Days after birth
O-Acetylcarnitine
Ratio of pup versus mother serum values
Days after birth
Homocarnosine
Ratio of pup versus mother serum values
Days after birth
2
7
13
18

**Figure S11. Selective overabundance of some non-essential amino acids in pup serum relative to mothers.** Changes in amino acids levels in the sera of pups with time after birth expressed as a ratio of the concentration in their mothers' sera. The horizontal dotted line indicates equivalence between levels in mothers and pups. As for Figures 5 and 6 of the main text, changes in abundance in the sera of pups with time after birth are expressed as a ratio of the concentration in their individual mothers' sera (ratio pup/mother; mean ± SE for each day). The days after birth labelled 18 are a combination of samples taken at 17, 18, or 19 days. The panels that follow present box and whisker plots of the data for each compound – see legends above for chart settings.

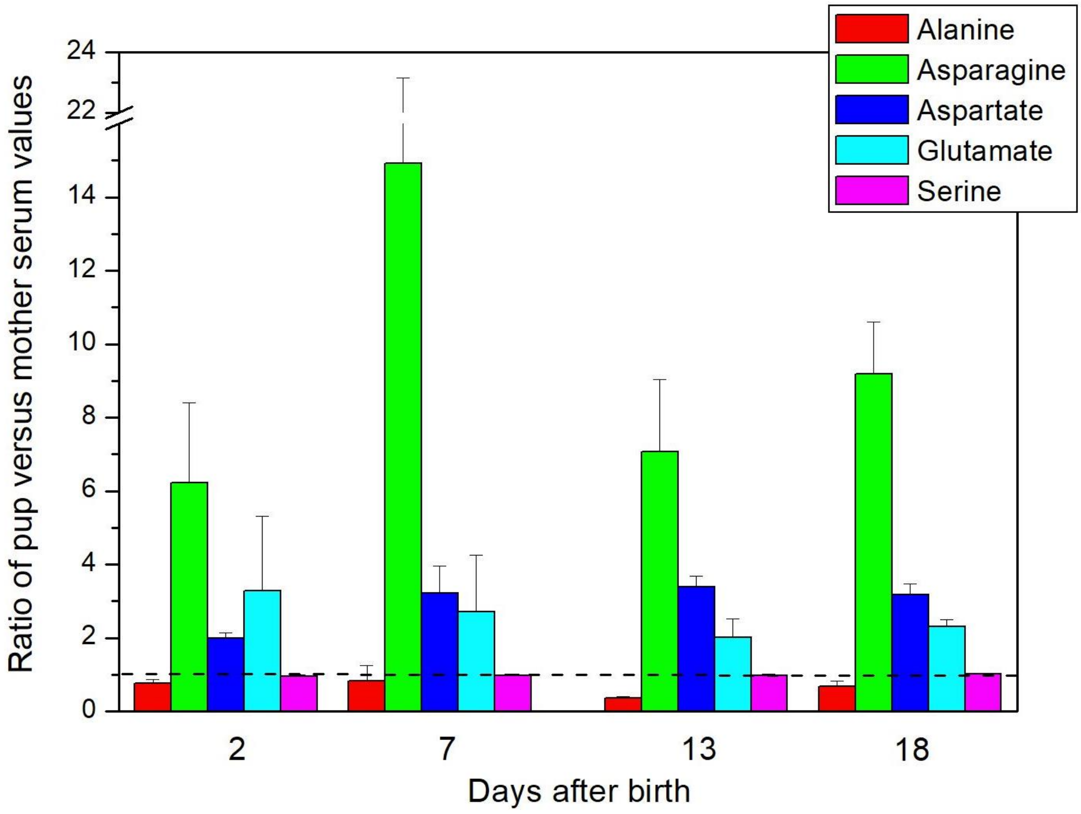

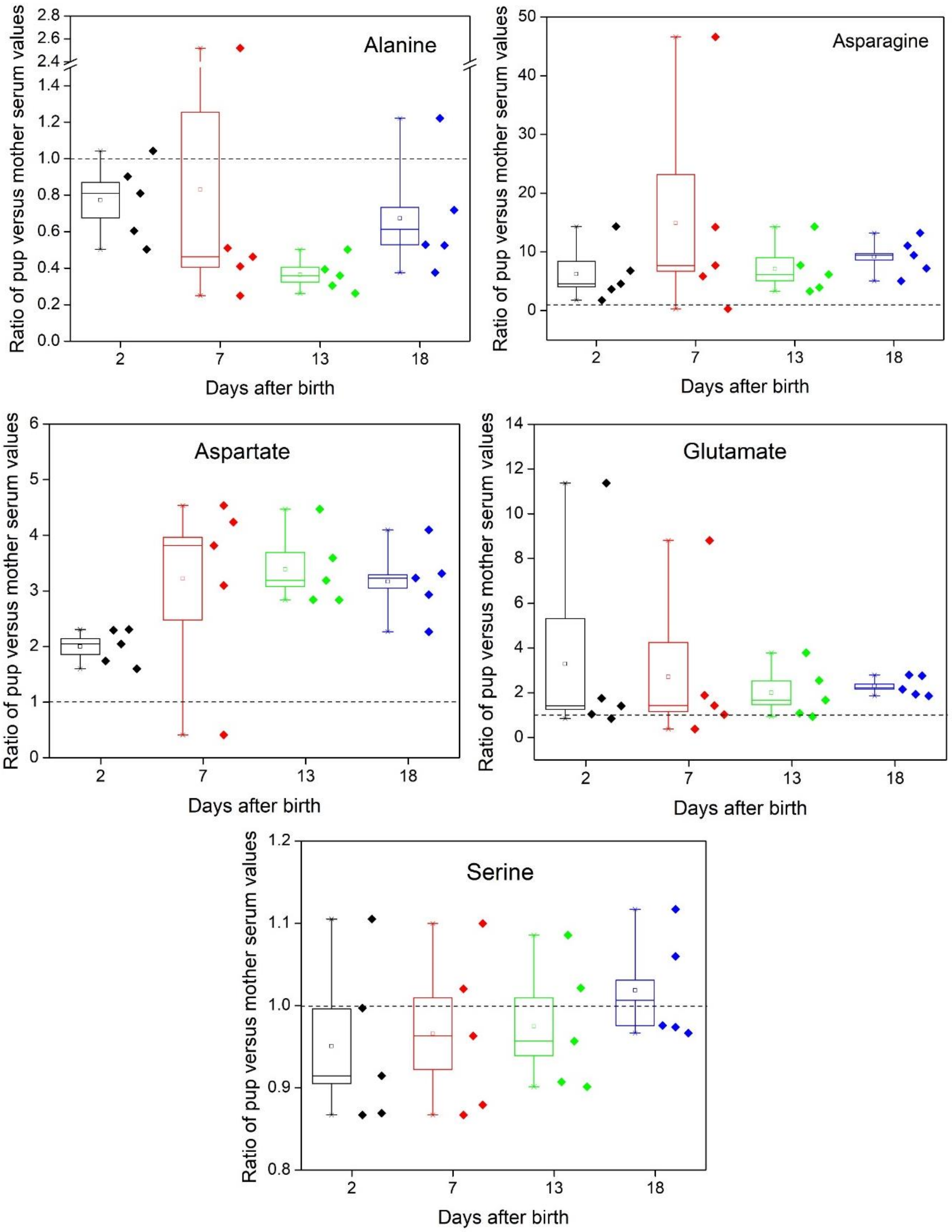


## References


1. Chong, J., et al., *MetaboAnalyst 4.0: towards more transparent and integrative metabolomics analysis.* Nucleic Acids Research, 2018. **46**(W1): p. W486-W494.
2. Lowe, A.D., et al., *Rapid changes in Atlantic grey seal milk from birth to weaning - immune factors and indicators of metabolic strain.* Scientific Reports, 2017. **7**.
